# Protein Motifs for Proton Transfers That Build the Transmembrane Proton Gradient

**DOI:** 10.3389/fchem.2021.660954

**Published:** 2021-06-15

**Authors:** Divya Kaur, Umesh Khaniya, Yingying Zhang, M. R. Gunner

**Affiliations:** ^1^ Department of Chemistry, The Graduate Center, City University of New York, New York, NY, United States; ^2^ Department of Physics, City College of New York, New York, NY, United States; ^3^ Department of Physics, The Graduate Center, City University of New York, New York, NY, United States

**Keywords:** proton transfer pathways, bacteriorhodopsin, photosystem II, bacterial reaction center, complex I, cytochrome c oxidase, Grotthuss mechanism

## Abstract

Biological membranes are barriers to polar molecules, so membrane embedded proteins control the transfers between cellular compartments. Protein controlled transport moves substrates and activates cellular signaling cascades. In addition, the electrochemical gradient across mitochondrial, bacterial and chloroplast membranes, is a key source of stored cellular energy. This is generated by electron, proton and ion transfers through proteins. The gradient is used to fuel ATP synthesis and to drive active transport. Here the mechanisms by which protons move into the buried active sites of Photosystem II (PSII), bacterial RCs (bRCs) and through the proton pumps, Bacteriorhodopsin (bR), Complex I and Cytochrome c oxidase (CcO), are reviewed. These proteins all use water filled proton transfer paths. The proton pumps, that move protons uphill from low to high concentration compartments, also utilize Proton Loading Sites (PLS), that transiently load and unload protons and gates, which block backflow of protons. PLS and gates should be synchronized so PLS proton affinity is high when the gate opens to the side with few protons and low when the path is open to the high concentration side. Proton transfer paths in the proteins we describe have different design features. Linear paths are seen with a unique entry and exit and a relatively straight path between them. Alternatively, paths can be complex with a tangle of possible routes. Likewise, PLS can be a single residue that changes protonation state or a cluster of residues with multiple charge and tautomer states.

## Introduction

Protons serve as substrate or product in many chemical and biological reactions. In proteins, protons often travel 10 Å or more from the surface to reach an active site. Proton gradients across the membranes of bacteria, mitochondria and chloroplasts contribute to the electrochemical gradients, ∆Ψ, used to store cellular energy (Mitchell, 1961; Rich, 2008; Nicholls, 2010; Gunner et al., 2013). The proton gradient can be generated by vectorial electron transfer, where reactants are oxidized and reduced on different sides of the membrane. Here the electrons cross the membrane, but the protons only move to or from the separated redox sites. In contrast, proton pumps transfer protons through the transmembrane proteins, requiring mechanisms to avoid downhill proton transfer.

Regardless of the mechanism a protein uses, it takes energy to build a proton gradient. The input energy is light in photosynthetic proteins (Cardona et al., 2012; Ge and Gunner, 2016; Cardona and Rutherford, 2019), redox reactions in the electron transfer chain (Kaila and Hummer, 2011), ATP hydrolysis (Vasanthakumar and Rubinstein, 2020) or the dissipation of the gradient of another ion (Fowler et al., 2015; Brandt, 2019). The protons flow down the electrochemical gradient then fuel processes such as ATP synthesis in F_1_/F_0_ ATPase (Walker et al., 1991; Futai et al., 2012; Yanagisawa and Frasch, 2017) and the active transport of ions and metabolites (Accardi and Picollo, 2010; Gunner et al., 2013).

To build the gradient, protons are transferred from the more negative, N-side of the membrane, where they are at lower concentration (higher pH) to the positive, P-side where they are at higher concentration (lower pH). The P-side is in the periplasm of bacteria, the outer membrane space of mitochondria and in the lumen on the inside of the chloroplast thylakoid membrane. The N-side is toward the bacterial cytoplasm, the mitochondrial matrix and the chloroplast stroma. The electrochemical gradient, ∆Ψ, is made up of the gradient of protons (the ∆pH) but also has contributions from other ions, adding to a voltage change, ∆V, across the membrane (Decoursey, 2003). The ∆Ψ across a given membrane determines the energy needed to push a proton uphill in the protein pumps described here or the energy liberated when protons run from P- to N-side as used for ATP synthesis.

Although we refer to “protons”, H^+^ does not travel alone. Rather it is associated with a water (hydronium, H_3_O^+^) or two water molecules as a Zundel cation (H_5_O_2_^+^) or as a larger, Eigen complex (H_9_O_4_^+^) (Agmon, 1995; Wraight, 2006; Farahvash and Stuchebrukhov, 2018). In proteins, the proton can also be bound to redox cofactors, to acidic or basic residues or trapped as a stabilized hydronium (Xu and Voth, 2006; Freier et al., 2011; Ikeda et al., 2017).

Protons move through a chain of oriented molecules by a Grotthuss proton transfer mechanism (Agmon, 1995; Cukierman, 2006; de Grotthuss, 2006; DeCoursey and Hosler, 2014). An active group in the middle of the chain is: 1) a hydrogen bond donor to the next group in the direction of proton transfer and 2) has a lone pair of electrons that is a hydrogen bond acceptor from the neighbor toward the proton input side. In the Grotthuss mechanism no proton moves more than one bond, as the proton acceptor takes ownership from the neighboring proton donor. However, overall the coupled transfers lead to a proton rapidly leaving the input side and appearing at the end of the chain. There are many reviews of the chemistry of proton transfer reactions as well as of proton transfer reactions in proteins (Hammes-Schiffer, 2001; Pomès and Roux, 2002; Blomberg and Siegbahn, 2006; Swanson et al., 2007; Knight and Voth, 2012; Ishikita and Saito, 2014; Miyake and Rolandi, 2015; Wikström et al., 2015; Sakashita et al., 2020).

Two requirements create barriers for Grotthuss proton transfers. First, the chain of hydrogen bonds between proton donors and acceptors needs to be pre-organized. Then, once the proton has transferred, the hydrogen bonds are arranged to return the proton back to the origin, not to move another proton in the same direction. The hydrogen bonded chain needs to fully reorient for the next proton to transfer, so overall proton flux is limited by this slow “hop and turn” process (Nagle and Morowitz, 1978).

*Vectorial proton coupled electron transfer.* Proteins such as PSII, cytochromes bc_1_ and b_6_f use vectorial electron transfer reactions where oxidation and reduction reactions are spatially separated to add to the proton gradient. Thus, oxidation occurs on the P-side, where protons are released because the loss of an electron lowers the oxidized product pK_a_ below the pH of the nearby compartment. Reduction occurs on the N-side, where reduction shifts the product pK_a_ to be higher than the compartment pH (Rich, 2008; Nicholls, 2010; Gunner et al., 2013; Gunner and Koder, 2017). Within the protein, a sequence of electron tunneling reactions pass the electrons 30 Å or more between the terminal electron donor and acceptor (Gray and Winkler, 2003; Moser et al., 2006). The interior electron transfer reactions are not coupled to gain or loss of protons. Thus, a proton gradient is generated without moving protons through the membrane by a redox loop mechanism as suggested by Mitchell (Mitchell, 1977). The intra-membrane, middle of these proteins are mostly non-polar side chains with few associated water molecules, so discourage proton transfer. However, as will be seen in the discussion of PSII and bRCs, the sites of final, proton coupled oxidation or reduction can be 10 Å or more from the surface, requiring long-range proton transfer to move the protons to the active site.

*Proton pumps.* The proton pumps include the well-studied, light-driven Bacteriorhodopsin (Balashov, 2000; Luecke, 2000), Complex I (Mathiesen and Hägerhäll, 2002; Hirst, 2013; Sazanov, 2014) and the heme copper oxidase (HuCuOx) family (Kaila et al., 2010; Lee and Ädelroth, 2013). To ensure protons move in the correct direction pumps require three elements. These are proton transfer paths, as found in vectorial electron transfer proteins. However, pumps need Proton Loading Sites, PLS, placed along the proton transfer path, and gates. PLS transiently change their proton affinity to load a proton when the gate is open to the N-side and releases it when it is open to the P-side. Pathway gating and proton loading must be synchronized to guard against energy dissipating proton transfer from P- to N-side.

This review will compare and contrast the residues that make up the proton transfer elements in three light activated proteins: Bacteriorhodopsin (bR) and the photosynthetic proteins, Photosystem II (PSII) and the purple non-sulfur photosynthetic bacterial reactions centers (bRCs) and in the proton pumps Complex I and Cytochrome c oxidase, which are the first and last protein in the erobic electron transfer chain.

### Overview of Proton Transfer Paths

*The role of the different residues in proton transfer paths.* The review will describe the residues found along proton transfer paths. Water is the quintessential Grotthuss competent molecule so water filled channels through protein structures often trace the proton transfer paths. However, some side chains can be a part of a proton transfer chain. Hydroxyl residues are well established in proton transfer paths, such as in Green Fluorescent Protein, GFP, which has a Ser on the short proton transfer path (Brejc et al., 1997; Donati et al., 2018). A neutral His has a proton on N_ε_ with a lone pair on N_δ_ (or the proton/lone pair swap positions). It can accept a proton from one side of the imidazole and donate a proton from the other side. His plays this role in proton transfer in the M2 proton channel (Wang et al., 1995) and in carbonic anhydrase (Tu et al., 1989). Histidine analogs have been used in synthetic electron coupled proton transfer chains that lead to the Grotthuss transfer of a proton over long distances (Odella et al., 2018; Odella et al., 2019). Ionized side chains cannot be both a hydrogen bond donor and acceptor as required for Grotthuss proton transfer (Ge and Gunner, 2016; Lazaridis and Hummer, 2017; Duster and Lin, 2019). Thus, deprotonated Asp^-^ and Glu^-^ are hydrogen bond acceptors but have no proton to donate, while protonated His^+^, Lys^+^ or Arg^+^ have no lone pairs to accept a proton. This review will show examples where acidic and basic residues are found as PLS in the proton transfer chain, serving as meta-stable intermediates that can cycle between loaded (protonated) and unloaded states. Polar residues such as Asn, Gln and Trp are found to anchor the hydrogen bond chain, but are unlikely be active elements in Grotthuss proton transfer chains (Hammes-Schiffer, 2001; Goings et al., 2020), while non-polar residue are insulators stopping water penetration and proton leaks.

*Linear vs. complex proton transfer paths.* As proton transfers have been investigated in different proteins, we have found they can take place via linear or complex paths. Linear paths, as defined here, have a single entry and exit and a well-defined road between them. There are limited branches, which never deviate far from the main path. Linear paths can often be identified in a protein structure that includes well resolved water molecules (Sharpe and Ferguson-Miller, 2008; Sazanov, 2015). Mutation of a single entry or exit residue can block proton transfer.

However, representative structures will be shown to reveal interior regions with tangled webs of polar and protonatable groups and many water molecules (Krammer et al., 2009; Cai et al., 2020; Khaniya et al., 2020). These complex proton transfer paths provide multiple choices for protons to follow. Here mutations of individual residue may lead to partial loss of activity, generating ambiguous results that neither fully confirm nor deny their role. The proteins reviewed here use linear and complex paths in different regions of the overall transfer of protons through the membrane.

### Proton Loading Sites

#### Type of Residues That Can Serve as PLS

A successful pump takes a proton through the protein from the N-side to the P-side, even though it is thermodynamically unfavorable. A PLS must transiently hold protons with gates open to the N-side and to be released to the P-side, synchronized with a turnover time of microseconds to milliseconds (Balashov, 2000; Kaila et al., 2010). The PLS is thus a residue or cluster of residues whose proton affinity changes dramatically between different reaction intermediates (Supplementary Material S2). The carboxylic acids, Asp, Glu and heme propionic acids are the most common PLS components in the proteins described here. They are found as single site PLS as well as PLS clusters. His and Lys are more often found coupled to acidic residues in clusters. H_3_O^+^, trapped between several acidic residues has been suggested to be part of PLS clusters (Freier et al., 2011; Kovalevsky et al., 2011; Supekar et al., 2016). However, the pK_a_ for Arg^+^ to lose a proton is as high as that of water or a hydroxyl side chain (Fitch et al., 2015). Thus, the protonated Arg can help stabilize the negative charge but is unlikely to lose a proton in a PLS.

*PLS clusters.* The PLS and complex proton transfer paths often have regions with many interacting, buried ionizable and polar residues (Lancaster et al., 1996; Kannt et al., 1998). For a PLS cluster with n protonatable residues there are n+1 charge states and 2^n^ microstates, which identify the number and distribution of protons (Gunner et al., 2020). The charge ranges from -N_acids_ (the number of acids) (assuming all bases are neutral) to +N_bases_ (number of bases) (assuming all acids are neutral). Tautomers are protonation microstates with the same charge but different proton locations. With m protons distributed over n binding sites in a PLS there are:

$$\frac{n!}{m!\left(n-m\right)!}$$ (1)

tautomers. The relative energies of the different tautomers determine the proton positions within the loaded and unloaded clusters. This review will describe examples of mechanisms by which PLS can change their protein affinity to load and unload protons.

## Model Systems

**FIGURE 1:**
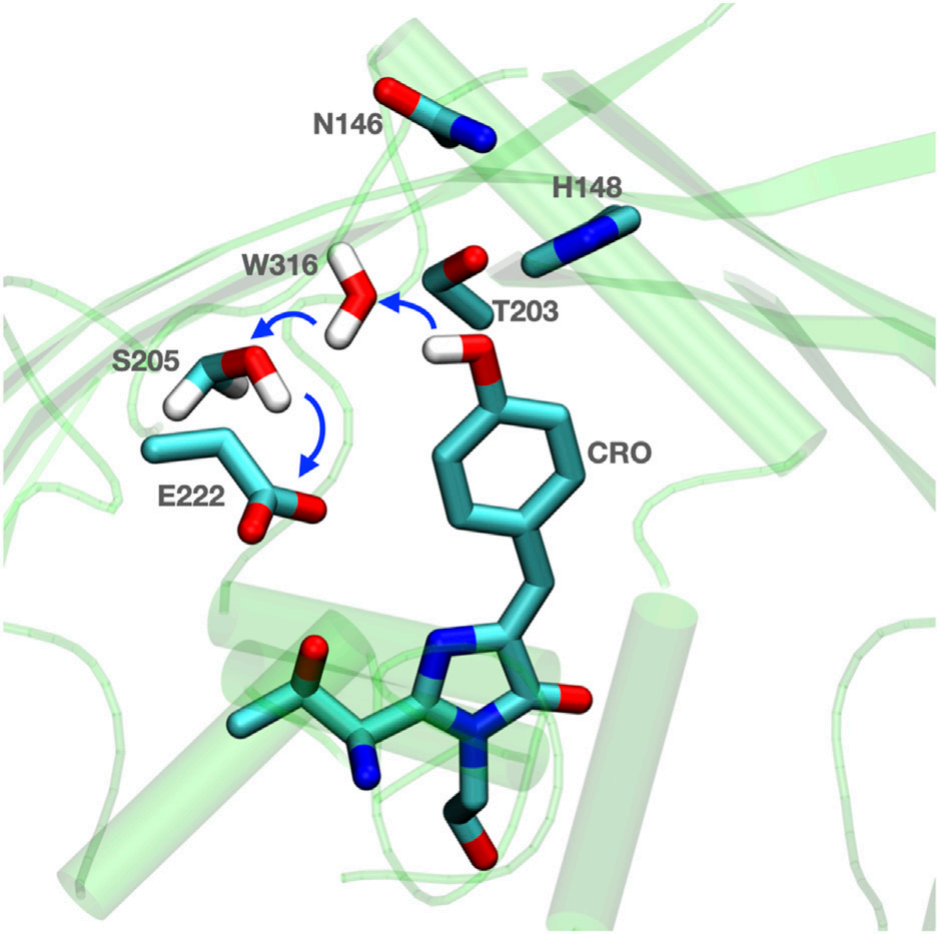
Residues are prearranged for Grotthuss proton transfer in GFP. Blue thick arrows show direction of proton transfer from chromophore (CRO) to E222 via water (W316) and S205. The surrounding H148 and N146 can help to anchor the active proton transfer path. The coordinates for *Equorea victoria* GFP are from PDB ID: 1EMA (Ormö et al., 1996).

*Green Fluorescent Protein* (*GFP*)*.* GFP provides a simple example of the role of side chains in and around the proton transfer path (Figure 1). It also shows how fast protons can transfer via a pre-organized Grotthuss competent chain (Brejc et al., 1997; Zimmer, 2009; Donati et al., 2018). GFP is well studied as it has revolutionized cell biology. When introduced into a genome it is co-expressed with a specific protein of interest and its characteristic florescence allows the targeted protein to be localized within a living cell. The chromophore in GFP is a photoacid that absorbs light in the near UV and emits in the green (Zhou and Han, 2018). The large Stokes shift results from the ground state absorption and excited state emission occurring from molecules with different charges. Thus, in the ground state the chromophore is the PLS, while Glu 222 is the proton acceptor when the chromophore proton affinity is diminished by excitation. In this system the path for rapid proton release must be ready to carry the proton away prior to relaxation of the chromophore (Chattoraj et al., 1996).

The proton is transferred through bound water and Ser 205 to Glu 222. A nearby His 148, Thr 203 and Asn 146 are on the outskirts of the proton transfer wire (Stoner-Ma et al., 2005; Di Donato et al., 2011). These provide a polar residue fence to pre-orient the hydrogen bond network. As the protein is light activated experiments can synchronize the protein for kinetic measurements to follow changes in the hydrogen bonding network. The proton arrives on the Glu in less than 10 ps (Donati et al., 2018). Vibrational spectroscopy shows that there are rapid changes in hydrogen bond orientation that precede proton transfer, presumably to fine tune the hydrogen bond connections for Grotthuss proton transfer. The kinetic transients are distinguished as rearrangements, which do not have a kinetic isotope effect, and proton transfers, which do (Di Donato et al., 2011).

*Gramicidin* (*gA*)*.* The gA channel is a proton and cation conducting channel that has been used to study proton transfer through a linear water wire, with no intervening side chains. gA is made of two short ß-strand peptides, capped on N- and C-terminal ends. The N-termini meet in the center of the membrane. In the ß-helix the side chains are to the outside, with sufficient space to hold a linear chain of ≈8 water molecules in the middle of the helix. gA is an antibiotic, allowing protons and other cations to cross, depolarizing the cell’s electrochemical gradient (Kelkar and Chattopadhyay, 2007; Li et al., 2016). The simplicity of this system has made it ideal for the experimental (Dorman and Jordan, 2004; Ryu et al., 2015; Paulino et al., 2020) and computational (Roux, 2002; Allen et al., 2004; Till et al., 2008; Lazaridis and Hummer, 2017; Zhang et al., 2020) studies of the Grotthuss proton transfer mechanism.

The water molecules in gA form hydrogen bonds with the two neighboring water molecules and with the amides of the surrounding peptides. The balance of the water-water and water-amide interactions determines the stability of the water wire, the stability of an excess proton within the wire and the barrier for flipping the wire orientation once a proton has translocated to transport another proton via the hop and turn mechanism. The rate determining step for transfer can be initially orienting the water molecules or flipping the oriented water dipoles to the correct direction (Pomès and Roux, 2002; Agmon et al., 2016; Bozdaganyan et al., 2019).

Recent computer simulations showed a rather substantial sensitivity of the water wire orientation to the force field and simulation method (Zhang et al., 2020). In molecular dynamics simulations, with a classical force field the water molecules are fully aligned within the channel with rare flips from one orientation to the other. In contrast, MD with a the Drude polarizable force field shows more disorganized water molecules. Monte Carlo sampling with a Continuum Electrostatic force field also show relatively disorganized water chain. Thus, the balance of the forces that determine the orientation of the water molecules are such that different simulation conditions induce different behavior.

Experiments have also supported a range of structures for water in the channel. The experimentally derived rate of proton translocation through the channel under a transmembrane voltage gradient appears to be diffusion limited (≈2 × 10^9^ s^−1^) even at pH 0 (Cukierman, 2000; Decoursey, 2003). The reorientation of the water chain is likely to be the rate determining step in Grotthuss proton transfer (Pomès and Roux, 1998). In the MD simulations with a classical force field the water chain flips its direction at ≈4 × 10^8^ s^−1^ and this process is faster with the Drude force field. Thus, the simulations are in general agreement with the measured rate of proton transfer. However, recent solid-state NMR studies show a well-organized water-wire with flip rates on the millisecond time scale, which would suggest very slow turnover for proton transfers. The NMR studies point to hydrogen bonds between water molecules and amides near the first and last turns of each ß-helix leading to this stability. Thus, despite the simplicity of its structure, gA remains a protein where our understanding of the channel water structure and the mechanism of proton transport remains incomplete.

## Bacteriorhodopsin

**FIGURE 2:**
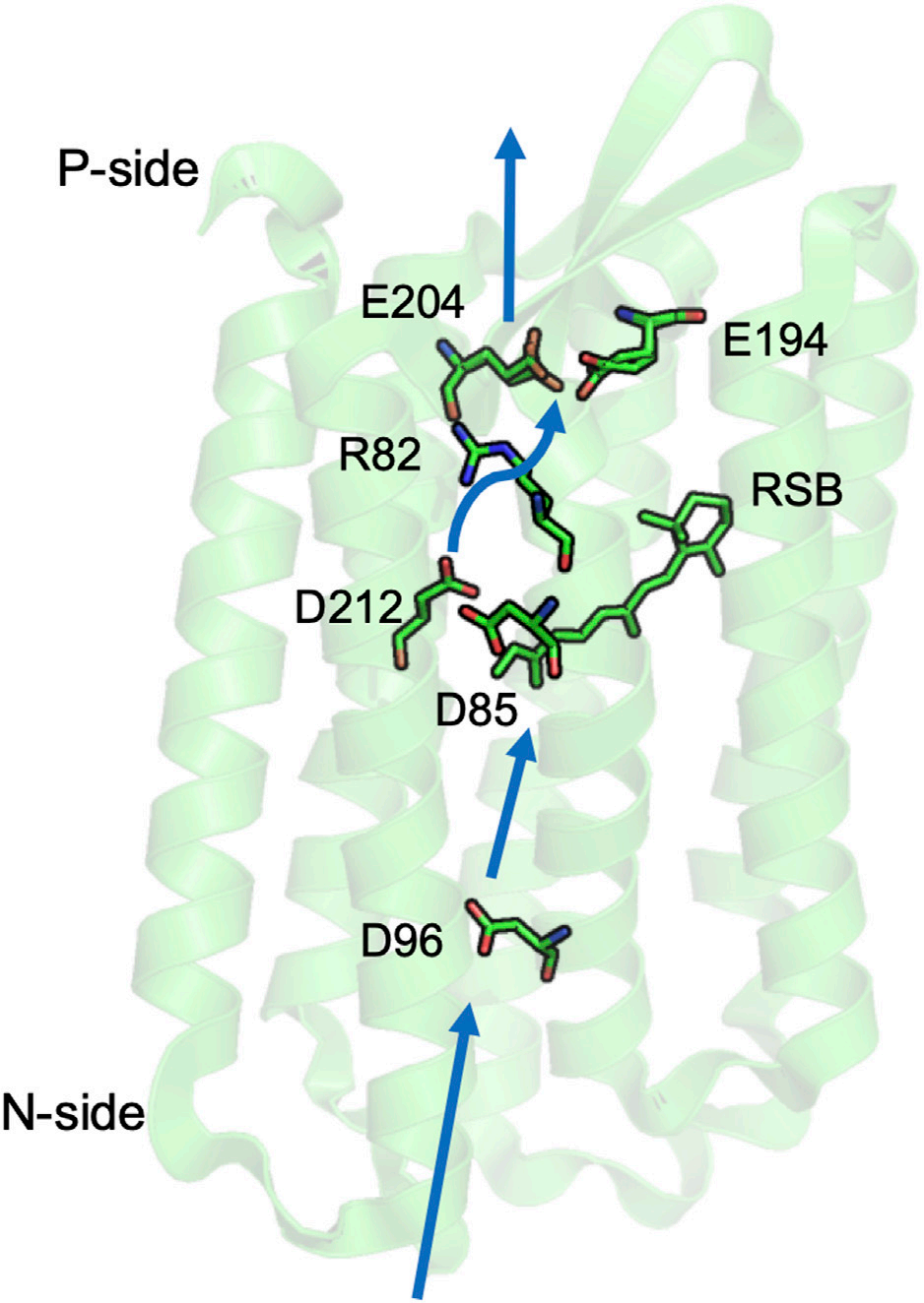
Key residues for proton transfer in bacteriorhodopsin. Three separated PLS are: the isolated D96; the central cluster D85, D212, and retinal Schiff base (RSB); and the exit cluster E194 and E204. Movement of R82 stabilizes unloading the central or exit clusters. Blue thick arrows show direction of proton transfer from N- to P-side by water mediated proton transfer paths. The coordinates for *Halobacterium salinarum* bR are from PDB ID: 5ZIM (Hasegawa et al., 2018).

bR is the simplest and best studied proton pump (Balashov, 2000; Baudry et al., 2001; Gunner et al., 2006; Lanyi, 2006; Lórenz-Fonfría et al., 2008; Clemens et al., 2011). The bacteriorhodopsin family uses retinal not chlorophyll based photoactivation to fuel proton or ion pumping (Figure 2). Absorption of a 568 nm photon initiates a reaction cycle that removes a proton from the cell interior (N-side) and releases one to the outside (P-side) adding to the proton gradient. The overall reaction is simply:

$${\text{H}}_{\text{N}-\text{side}}^{+}\;+\text{ hv }\to {\text{ H}}_{\text{P}-\text{side}}^{+}$$

The proton transfers are driven by the light induced conformational changes of the retinal from all trans to 13-cis, which is coupled to small changes in the helical packing in the protein core. There are three PLS in bR that illustrate different mechanisms to control PLS protonation and the connection to the proton transfer path.

*Characterization of proton transfer intermediates.* In light activated proteins such as GFP, bR, PSII and bRCs experiments can follow the sequential reaction mechanism since the flash of light used to start the reaction synchronizes the population. Time resolved visible and IR spectroscopy, combined with site directed mutations allow assignment of kinetic features to individual residues. bR intermediates were identified with the proton resting on acidic amino acids making up three well separated PLSs, each with metastable intermediates with different ionization states (Balashov, 2000; Lórenz-Fonfría and Kandori, 2009; Lórenz-Fonfría et al., 2011; Lórenz-Fonfría and Heberle, 2014). These intermediates clearly demonstrate the role of transient resting places for protons. Changes occur in times ranging from the picosecond transitions that trap the photon’s energy in the isomerized retinal to the milliseconds required to complete the full photocycle.

In bR, it is possible to crystallize protein trapped in different intermediates by a combination of mutation and temperature changes (Edmonds and Luecke, 2004; Hirai et al., 2009; Wickstrand et al., 2015). Simulations using these structures have shown that the calculated equilibrium proton distribution changes between intermediates as expected (Bashford and Gerwert, 1992; Spassov et al., 2001; Onufriev et al., 2003; Song et al., 2003; Song and Gunner, 2014). More recent time resolved crystal structures have been obtained using X-ray free electron lasers (XFEL) (Nogly et al., 2018; Wickstrand et al., 2019). These structures show many of the motions of water molecules and side chains and helices seen in earlier trapped structures. However, as the XFEL structures are not in deeply trapped intermediates, the dynamic structures provide additional information. However, as the transitions between photocycle intermediates are not all well separated in time, the XFEL structures each contain a mixture of states.

### bR Demonstrates the Character of Simple and Cluster PLS

*The central cluster tautomer shift.* The central cluster consists of three residues: the retinal Schiff base (RSB), Asp 85 and Asp 212, which binds one proton on the RSB in the ground state (RSBH^+^: Asp 85^-^: Asp 212^-^). Light absorption leads to isomerization of the retinal, which rotates the RSB from facing the P-side to the N-side, leaving the proton on Asp 85 (M state: RSB: Asp85H: Asp 212^-^). This transition demonstrates a feature of a cluster PLS, as it moves between states with different proton distributions (tautomers) while retaining the same number of protons. The redistribution of the proton coupled to the retinal isomerization serves as a gate as it changes the direction of proton access. Thus, a proton will be passed from the trans-RSB to Asp85 toward the P-side, while later a proton is bound to the 13-cis RSB from the N-side (Bondar et al., 2007; Clemens et al., 2011; Wolter et al., 2013). The retinal returns to the P-side facing trans isomer only after it has bound the proton (Balashov, 2000).

*A complex PLS can trap a proton on multiple sites.* The complex exit cluster PLS, with Glu 194 and 204, has multiple tautomers for the proton loaded state. IR spectroscopy (Daldrop et al., 2018) and simulations (Bashford and Gerwert, 1992; Spassov et al., 2001; Phatak et al., 2008) support a protonated water stabilized by the two anionic glutamic acids, while the proton can also be trapped by a hydrogen bonded pair with one acid protonated and a water nearby (Song et al., 2003; Phatak et al., 2008). An advantage of using a cluster PLS is that it can use the multiple ways to store the proton to be less sensitive to mutation. If one of the Glu is mutated to an Asp the cluster is no longer properly positioned to trap a hydronium so the water cation IR signature is lost. The proton is now trapped on an acid, thereby retaining PLS function (Balashov, 2000; Gerwert et al., 2014).

*An isolated acidic PLS requires hydration to lose its proton.* Asp 96 on the N-side of bR plays a key role in proton transport (Miller and Oesterhelt, 1990). Asp 96 is an isolated PLS, as it is not in a cluster with other protonatable residues and has few hydrogen bonding opportunities to residues beyond Thr46 in the neighborhood. In the neutral, unloaded structure, there are few nearby water molecules and the acid is very stable in its neutral, loaded state (Gerwert et al., 2014; Wolf et al., 2014). A combination of time resolved IR and MD simulations show that isomerization of the retinal, 10 Å from Asp96, and the transfer of the proton from RSBH^+^ to the nearby Asp 85 leads to formation of a linear water chain on the N-side (Freier et al., 2011). The water molecules provide a proton transfer path, and also stabilize the negative charge on Asp 96 so that it can release a proton to the RSB near the end of the photocycle. Thus, a single site PLS cannot change its proton affinity by small movements of the polar and charged groups. Here flooding the site with water is required to both stabilize the charged Asp^-^ and to open the gate for proton release by connecting it to the proton transfer path.

## Photosystem II

**FIGURE 3:**
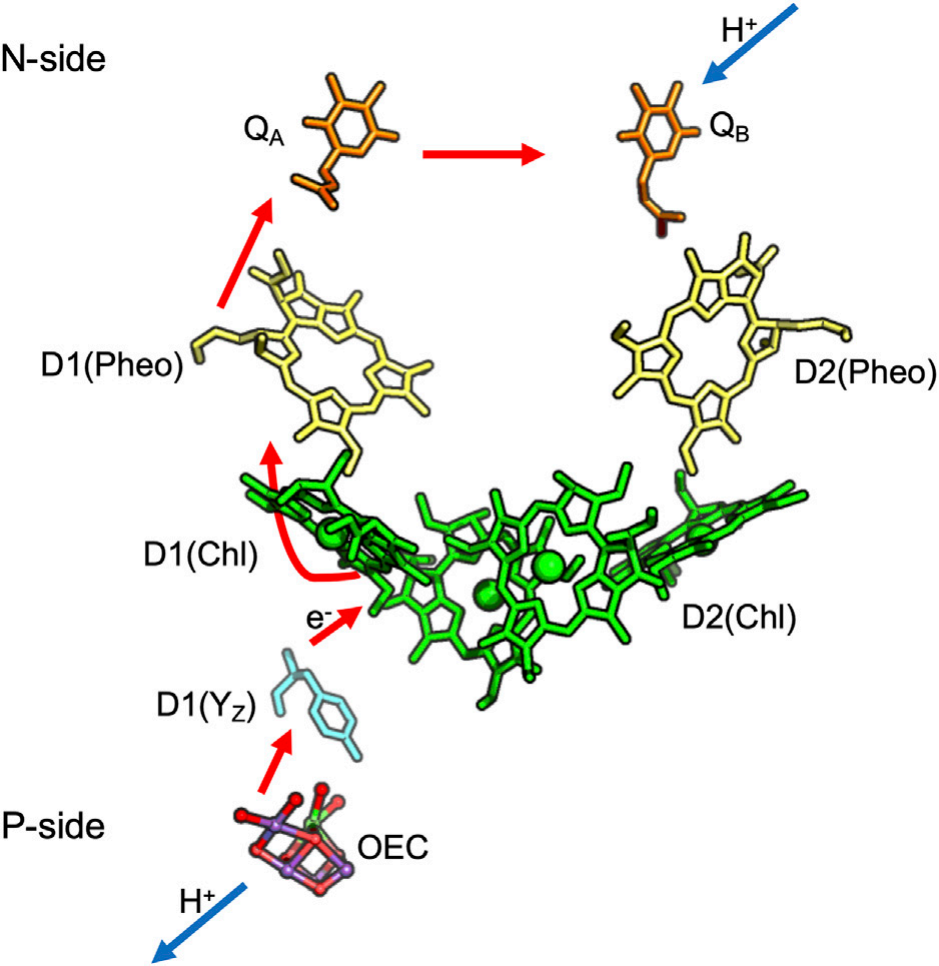
Cofactors in PSII: Q_A,_ Q_B_ are plastoquinones, Pheo is Pheophytin; Chl is Chlorophyll. D1 and D2 are the protein subunit that binds each cofactor. Red arrows show electron transfer from the OEC to Q_A_ and Q_B_ while blue lines show proton uptake from the stroma to Q_B_ and release from the OEC to the lumen. The coordinates for cyanobacteria *Thermosynechococcus vulcanus* are obtained from PDB ID: 3ARC.

PSII (Umena et al., 2011) is a multi-subunit protein pigment complex present in the thylakoid membrane of plants and cyanobacteria (Cox et al., 2013; Vinyard and Brudvig, 2017; Pantazis, 2018). The reaction is initiated by chlorophyll excitation with a 680 nm photon. Water is the primary electron donor and plastoquinone, PQ, is the final electron acceptor (Figure 3) (McEvoy and Brudvig, 2006). The overall reaction is:

$${2\text{H}}_{2}\text{O }+\;4\text{h}v{\;+\;2\text{PQ }+\;4\text{ H}}_{\text{stroma}}^{+}\;\to {\text{ O}}_{2}{\;+\;2\text{PQH}}_{2}{\;+\;4\text{ H}}_{\text{lumen}}^{+}$$

**FIGURE 4:**
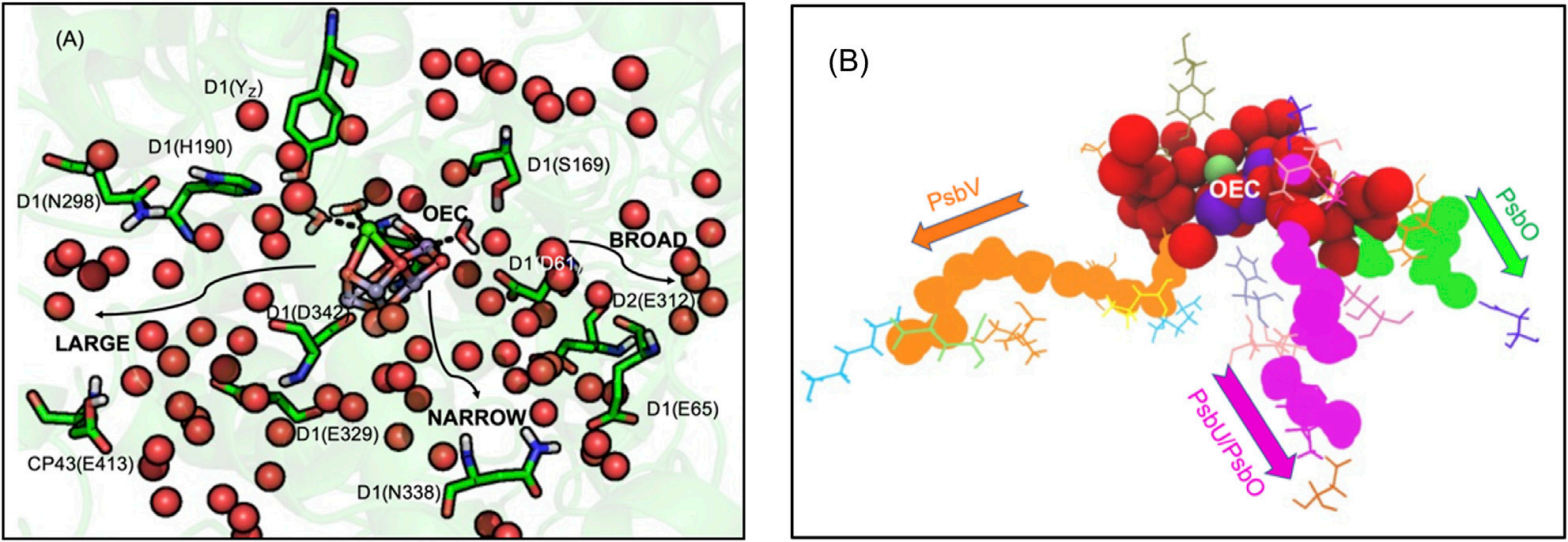
**(A,B)**. **(A)** Water channels around the Oxygen Evolving Complex (OEC) of PSII. Water spheres (red) and amino acid residues highlighting the directions toward the large, broad and narrow channels (Vassiliev et al., 2012; Vogt et al., 2015). The notation for each residue is subunit-residue type, number. Residues in large channel: Y_Z_, D1-H190, N298, E329, D342 (OEC primary ligand), CP43-E413. Narrow channel: D1-S169, N338. Broad channel: D1-D61, E65, D2-E312. **(B)** Waters extending from the OEC to the N-side lumen. Red spheres show highly interconnected water molecules extending ≈10 Å from the OEC. The structure then resolves to form three separated water filled channels: large (orange), narrow (magenta) and broad (green). Large channel extends from O1 of OEC to PsbV, narrow channel extends from O4 to PsbU/PsbO while broad channel extends from O5 to PsbO. See Supplementary Figure S1 for a more detailed view of the OEC. Coordinates from one MD snapshot initiated from *Thermosynechococcus vulcanus* PDB ID: 4UB6.

The Oxygen Evolving Complex (OEC), an inorganic Mn_4_CaO_5_ cluster, catalyzes water oxidation following four sequential oxidations of the cluster (through five S-states) releasing O_2_ (Suga et al., 2019). The OEC is ≈20 Å from the surface, requiring paths for water entry and O_2_ and proton release. The region around the OEC is filled with water molecules that separate into three discrete water-filled channels moving to the lumen (Figures 4A,B) (Vassiliev et al., 2012; Vogt et al., 2015). On the electron acceptor side, Q_B_ in PSII is quite close to the stroma requiring only a short proton transfer path to bring in protons (Saito et al., 2013).

### Proton Paths to the P-Side Near the OEC in PSII

*Three water filled paths.* Proton transfer paths combine Grotthuss competent water molecules and residues with protonatable groups to serve as transient proton loading sites. The proton release paths in PSII are dominated by water molecules, requiring minimal assistance from residues. There are three identified water filled channels leading from the OEC to the lumen (Figure 4A) (Vassiliev et al., 2012; Vogt et al., 2015). The narrow channel originates from one side of the Mn4 water ligands and extends through the PsbU/PsbO subunits (Figure 4B). The broad channel originates from the other side of Mn4, extending to the PsbO subunit, while the large channel originates from the OEC Ca water ligands leading to the PsbV subunit (Vassiliev et al., 2012; Vogt et al., 2015). These channels can provide paths for the transfer of the four product protons and O_2_ to the lumen and entry of the two substrate water molecules.

*Which path does the proton take?* With so many choices, an open question is which channel is best suited for proton transfer to the surface. Various simulation techniques have explored the nature of the water channels. Molecular dynamics investigations (Vassiliev et al., 2012) and quantum chemical studies (Retegan et al., 2016) favored the narrow channel for substrate water delivery. QM/MM studies (Saito et al., 2015) supported proton transport through the narrow channel. Continuum electrostatics calculations considered the proton affinity of residues lining the broad channel, finding increasing proton affinity, lowering the barrier for proton transfer, nearer the channel exit (Ishikita et al., 2006). Steered MD calculations (Vassiliev et al., 2012) found the large channel favorable for O_2_ transport and the narrow channel for substrate water delivery. However, other experimental and computational studies favored the large channel for proton transport (Chrysina et al., 2011; Nakamura et al., 2014; Sakamoto et al., 2017) or for substrate water delivery (McEvoy and Brudvig, 2004; Isobe et al., 2015; Shoji et al., 2015; Ugur et al., 2016; Kim and Debus, 2017). Thus, despite experimental and computational studies, a consensus for the role of each channel is yet to be established (Pantazis, 2018).

While earlier studies focused on individual linear paths, network analysis provides a somewhat different view of the connectivity of the water networks near the OEC (Kaur et al., 2021). These studies indicate that beyond ≈10–12 Å from the cofactor the three paths do become well separated as indicated by inspection of the structures. However, closer to the OEC all water molecules are highly interconnected. A proton from any of the Mn terminal water ligands or any of the oxygens that bridge the OEC Mn (except O2 and O3) can find its way to any of the three channels (Figure 4B) (Kaur et al., 2021). Comparing the proton affinity of H_3_O^+^ placed on individual water molecules in the three separated paths shows the broad channel as being more hospitable to the positive charge as suggested earlier (Ishikita et al., 2006; Bondar and Dau, 2012).

*Mutations and time resolved IR difference spectroscopy support a complex proton transfer path near the OEC.* The question is how to characterize a highly interconnected proton transfer path dominated by water molecules. FTIR-difference spectra followed through the cycle of reactions that lead to oxygen evolution shows changes in an extensive hydrogen bonding network around the cofactor. For example, FTIR-difference spectra shows a carboxylate peak (near 1,747 cm^−1^) whose proton affinity decreases in the step in the OEC oxidation cycle where a proton is not released to the lumen (S_1_ to S_2_) (Debus, 2015). This feature is lost when mutations are made of residues separated by ≈20 Å including D1-Asp61Ala, D1-Glu65Ala, D2-Glu312Ala, D1-Arg334Ala, D1-Glu329Gln (Figure 4). Mutation of each of these residues disrupts the hydrogen bond network and blocks or slows O_2_ evolution (Service et al., 2010; Debus, 2014). All of these residues are found in the network analysis that reveled the connections of all water molecules near the OEC (Kaur et al., 2021).

**FIGURE 5:**
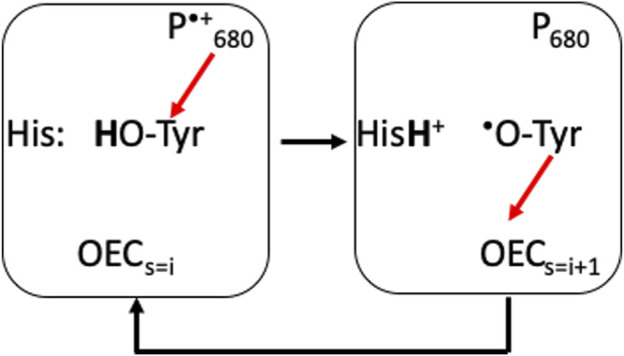
D1-His 190 is an example of a PLS supporting transient redox cycling of Y_Z_ (D1-Tyr 161), which is an intermediate electron donor/acceptor in the PSII electron transfer chain. Red arrows show electron transfers. Y_Z_ is an electron donor to the oxidized P_680_^•+^ (≈11 Å away) and acceptor from the OEC (≈5 Å away). The pK_a_ of the oxidized Tyr is <0, while it is 9.8 when it is reduced. His 190 ensures that the proton is captured when the Tyr is oxidized and returned when the Tyr is reduced. As Y_Z_ and His 190 are hydrogen bonded together, the proton simply moves between the two residues and does not escape (Ishikita and Saito, 2014).

*PLS used for proton coupled electron transfer near the OEC.* PSII provides an example of the use of a PLS to stabilize the redox reactions of an intermediate on a longer electron transfer chain. Through the S-state cycle the redox active Tyr161, Y_z_, is the electron donor to the oxidized chlorophyll, P_680_^•+^. Y_z_^•+^ is then reduced by the OEC (Figure 5) (Lavergne and Junge, 1993). The pK_a_ of an oxidized Tyr is -2 (Tommos and Babcock, 2000), so Y_z_^•+^ will lose its proton. D1-His 190 serves as a PLS, trapping the proton for the microseconds to several milliseconds that Y_z_ is oxidized (Figure 5) (Rappaport et al., 1994). This His has a low enough proton affinity that it is neutral in the ground state, yet its proton affinity is higher than the oxidized Y_z_^•^. The protein must block the proton from being lost to the lumen from the His. A tight hydrogen bond between the Tyr and the His helps as does the presence of polar, but non-proton conducting residues such as D1-Asn 298 surrounding the pair (Saito et al., 2011). The proton is shuttled between the Tyr cofactor and its adjacent PLS, never moving in or out of the protein, while the electron is passed from the OEC to P_680_ via Y_Z_ (Figure 5) (Saito et al., 2011; Ishikita and Saito, 2014).

*Gates in proton transfer pathways in proteins that are not pumps.* A gate along proton transfer path guards against unwanted proton transfers. An inhibitory gate can be identified in PSII, where a chloride ion helps to keep the entrance to the broad channel open by preventing a salt bridge between D1-Asp 61 and D2-Lys 317. Experiments show chloride depletion blocks the advancement of S-state transition beyond S_2_ (Ono et al., 1986; Pokhrel and Brudvig, 2014). Simulations show depletion of chloride leads to formation of a salt bridge between D1-Asp 61 and D2-Lys 317 hindering proton loss (Rivalta et al., 2011; Amin et al., 2016; Kaur et al., 2019).

*Fences support a proton path*. The sides of the water filled channels contain residues such as Asn and Arg (e.g., D1-Asn 87 and CP43-Arg 357). These cannot participate in Grotthuss proton transfers, nor are their pK_a_ in a range that would let them be PLS. Rather, these residues can anchor, the hydrogen bond connections, orienting the water molecules. One example that has been investigated is D1-Asn 298 near the OEC. Mutating this residue influences oxygen evolution (Kuroda et al., 2014) and the FTIR spectrum of the OEC network (Nagao et al., 2017; Chrysina et al., 2019). Simulations show the Asn changes the orientation of its side chain amide dipole in the transition from S_2_ to S_3_ leading to rearrangement of the hydrogen bond network (Chrysina et al., 2019).

## Bacterial Reaction Center

The reaction center, bRC, of the purple non-sulfur bacteria is the first membrane protein whose structure was solved at atomic resolution (Deisenhofer et al., 1985). RCs are light activated proteins so as with GFP, bR and PSII, time resolved measurements allow individual steps in the series of electron and/or proton transfer reactions to be monitored by time-resolved spectroscopy, showing the individual steps in the reactions (Okamura et al., 2000; Wraight, 2006).

bRCs and PSII are Type II reaction centers where a fully reduced, QH_2_ is the final product. The quinol dissociates into the membrane to serve as the substrate of the b_6_f complex in oxygenic photosynthesis and the bc_1_ complex in bacteria (Cardona et al., 2012; Cardona and Rutherford, 2019). The D1 and D2 subunits of PSII are related to the L and M subunits in bRCs (Raymond and Blankenship, 2004). The bacterial systems use a photon, in the range of 860–960 nm. Thus, they do not have enough energy to carry out the PSII reaction, which uses a 680 nm photon to fuel the uphill transfer of electrons from water to quinone (Heathcote et al., 2002). The primary electron donor in bRCs is periplasmic (P-side) cytochrome c. The redox reactions of cytochrome c are not coupled to proton binding/release. The overall reaction is:

$${2\text{cyt c}}_{\text{P}-\text{side}}^{2+}\;+\;2\text{h}v{\;+\text{ UQ }+\;2\text{H}}_{\text{N}-\text{side}}^{+}\;\to {\;2\text{cyt c}}_{\text{P}-\text{side}}^{3+}{\;+\;2\text{UQH}}_{2}$$

**FIGURE 6:**
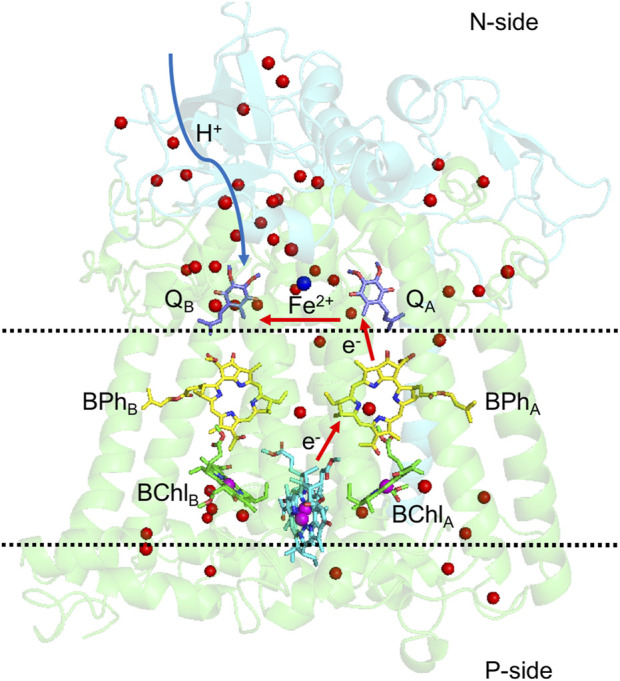
Structure of bacterial photosynthetic reaction centers of *Rhodobacter sphaeroides*. L and M subunits in green, H subunit in cyan. Red arrows follow the electron transfer path, and the blue arrow is the path protons travel from the N-side to Q_B_. Water molecules in the structure are shown as red balls. The region between the two dashed lines has few water molecules or polar residues, which discourages proton transfer across the protein. The two quinones are still within the lipid membrane, but in a region of the protein with multiple polar residues and water molecules to transport protons. Coordinates are from PDB ID: 1AIG.

In contrast to PSII, which has a very short distance to the N-side, bRCs have an H subunit, capping the N-side of the protein, requiring a much longer path for the protons to reach the Q_B_ site (Figure 6).

*Quinones as a model redox coupled proton transfer reactant.* Electrons move one at a time between cofactors in proteins generating free radical intermediates. While some cofactors such as chlorophylls, hemes, iron sulfur clusters and Tyr are stable one electron redox cofactors, unpaired electrons are often sources of toxic reactive oxygen intermediates (Weisz et al., 2017). Quinones function as single electron donors/acceptors within proteins, but accumulate two electrons and protons (Paddock et al., 2003; Müh et al., 2012). These lipid soluble cofactors thus transport electrons from many proteins including PSII, bRCs and complex I described here to the bc_1_ complex in mitochondria and bacteria or b_6_f complexes in chloroplasts as electrons move down the electron transfer chains.

As quinones cycle between oxidized quinone (Q), semiquinone (Q^•-^) and fully reduced quinol (QH_2_) their proton affinity changes. The quinone pK_a_ is <0, it is <5 for the semiquinone (Zhu and Gunner, 2005; Gunner et al., 2008; Hasegawa et al., 2017). Hence, the anionic semiquinone is often the stable state within the protein. However, the generation of Q^•-^ can trigger proton binding to nearby residues (Graige et al., 1996; Abresch et al., 1998; Alexov and Gunner, 1999; Paddock et al., 2003). The pK_a_s for the fully reduced quinol is >10 so the second Q_B_ reduction is coupled to binding two protons to the cofactor. The difference in the hydrogen bonding pattern for the two quinone carbonyls and two quinol hydroxyls promote quinone dissociation in PSII (Shevela et al., 2012; Saito et al., 2013). As will be described below, in Complex I quinone reduction leads to a large conformational change that trigger proton pumping through distant pathways (Gupta et al., 2020; Gutiérrez-Fernández et al., 2020; Kampjut and Sazanov, 2020).

The quinone electrochemistry is modified within the protein, but the underlying proton affinity of each redox state in the isolated compound strongly influences the order in which protons are bound. The sequence of reactions in bRCs (Graige et al., 1996) and likely in PSII is: 1) Q_B_ is first reduced to the anionic semiquinone. The negative potential causes protons to be bound to nearby amino acids (Okamura and Feher, 1992; Wraight, 2006; Gunner et al., 2008); 2) uphill protonation of the semiquinone is the rate-determining step preceding the second reduction; 3) reduction is followed by binding a second proton and release of the quinol.

**FIGURE 7:**
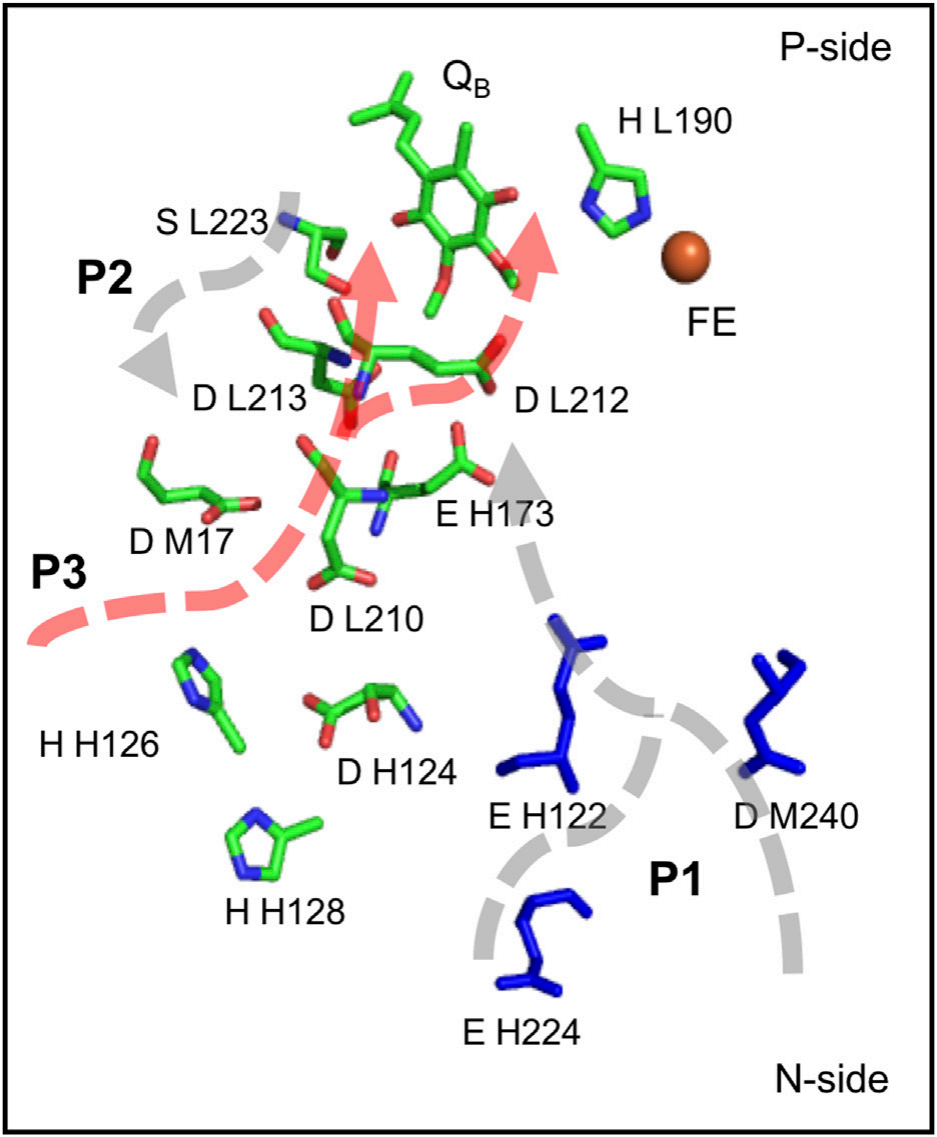
Three proposed paths for the two protons from the surface to the Q_B_ site (Abresch et al., 1998), showing key residues on each path including H-His 126, H-His 128, H-Asp 124, L-Asp 210, M-Asp 17, H-Glu 173, L-Asp 213, L-Ser 223 and L-Glu 212 (Okamura et al., 2000; Paddock et al., 2003; Wraight, 2004). Coordinates from *Rb. sphaeroides bRCs* PDB ID: 1AIG.

*Proton transfer pathways to Q*_*B*_ *in bRCs.* The proton transfer paths to Q_B_ have been well studied (Abresch et al., 1998; Okamura et al., 2000). These are different than those described above near the OEC, as residues play a much larger role in the network. However, they are similar in that there is a tangled complex of proton transfer paths. There is a large number of acidic and basic residues buried in the protein near Q_B_ that influence the electrochemistry of the quinone and provide paths for proton transfer (Figure 7) (Sebban et al., 1995; Lancaster et al., 1996; Abresch et al., 1998; Alexov and Gunner, 1999; Rabenstein et al., 2000). FTIR difference spectra obtained on Q_A_ and Q_B_ reduction shows broad features characteristic of a polarized, interconnected hydrogen bonded network of water molecules and amino acids around the two quinones (Breton and Nabedryk, 1998). L-Asp 210 and Asp 213 may share a proton in the ground state, serving as a PLS. Protonating one of the acids removes a negative charge, stabilizing the semiquinone Q_B_^•-^ and keeping a proton available for passage to the quinone itself (Lancaster et al., 1996; Alexov and Gunner, 1999; Ishikita et al., 2003). The mutants L-Asp213Asn and L-Ser223Ala slow the rate of this reaction, with the mutation of L-Asp 213 having a bigger impact (Paddock et al., 1994; Paddock et al., 1995). In the absence of L-Asp 213, H-Glu 173 may provide an alternative location for the proton in this extended PLS (Paddock et al., 2003). Thus, this web of acidic residues combines the functions of a cluster PLS and complex proton transfer path.

L-Glu 212, which is a protonated PLS in the ground state, provides the second proton to Q_B_ (Wraight, 2004). The pK_a_ of the Glu is ≈10, trapping a proton near the quinone in the ground state ready when needed (Kleinfeld et al., 1984; Okamura and Feher, 1992). The mutant L-Glu212Gln does not affect the delivery of the first proton, supporting this site being neutral in the presence of Q_B_^**-**^. However, the transfer of the second proton is totally blocked, indicating L-Glu 212 is a unique single site PLS (Paddock et al., 1989; Shinkarev et al., 1993; P. H.; McPherson et al., 1994; Okamura et al., 2000; Wraight, 2004).

*Three possible paths.* The complex web of acidic and other polar residues near Q_B_ leads to the question of what is the route for proton transfer from the N-side surface to the quinone binding site. The crystal structures reveal three likely paths (Figure 7) (Abresch et al., 1998). The longest path, P1, is ≈20 Å long. It enters the protein near H-Asp 224 or M-Asp 240 and passes to L-Glu 212, which provides the second proton to Q_B_. P2, also ≈20 Å long, starts near M-Tyr 3 and moves via H-Glu 173 to L-Asp 213, which donates the first proton to Q_B_. P3 is the shortest path, with only ≈7 Å between L-Asp 213 and the surface M-Asp 17 with one water molecule in the middle (Abresch et al., 1998).

*Surface PLS as a proton collection site.* RCs also have a well characterized external cluster near the entrance to P3 made up of H-Asp 124, H-His 126, H-His 128. The cluster is a proposed proton collection site (Utschig et al., 1998; Paddock et al., 1999; Okamura et al., 2000) (Figure 7). Zn^2+^ or Cd^2+^ bind here and slow proton transfer to Q_B_. Clusters of protonatable groups near the surface of proton transfer paths are found in other proteins. A similar proton accumulation site is found in the D-channel (Cai et al., 2018). The broad channel in PSII exits to a cluster of surface acidic residues that can trap the released proton (Bondar and Dau, 2012; Kaur et al., 2021).

P3 appears to carry protons in wild-type bRCs. Mutation of L-Asp 210 and M-Asp 17, have a larger impact when Zn^2+^ or Cd^2+^ are present, showing an additive effect of multiple changes to this pathway. However even with P3 blocked, protons still enter to Q_B_, indicating that other routes can serve as pathways, but with slower transfer rates (Okamura et al., 2000). Thus, in the tangled potential proton transfer network, multiple paths are possible, but some are preferred.

## NADH-Ubiquinone Oxidoreductase (Complex I)

Complex I is the first and the largest protein in the erobic respiratory electron transfer chain of bacteria and mitochondria (Brandt et al., 2003; Hirst, 2013; Sazanov, 2015; Kaila, 2018). Within the protein electrons are transferred from NADH to a flavin and through a series of Iron Sulfur (FeS) complexes to a quinone (Verkhovskaya et al., 2008; Efremov et al., 2010; Hirst, 2013; Zickermann et al., 2015). The overall reaction is:

$${\text{NADH }+\text{ H}}^{+}{\;+\text{ Q }+\;4\text{H}}_{\text{N}}^{+}\to {\text{NAD}}^{+}{\;+\text{QH}}_{2}{\;+\;4\text{H}}_{\text{P}}^{+}\;$$

**FIGURE 8:**
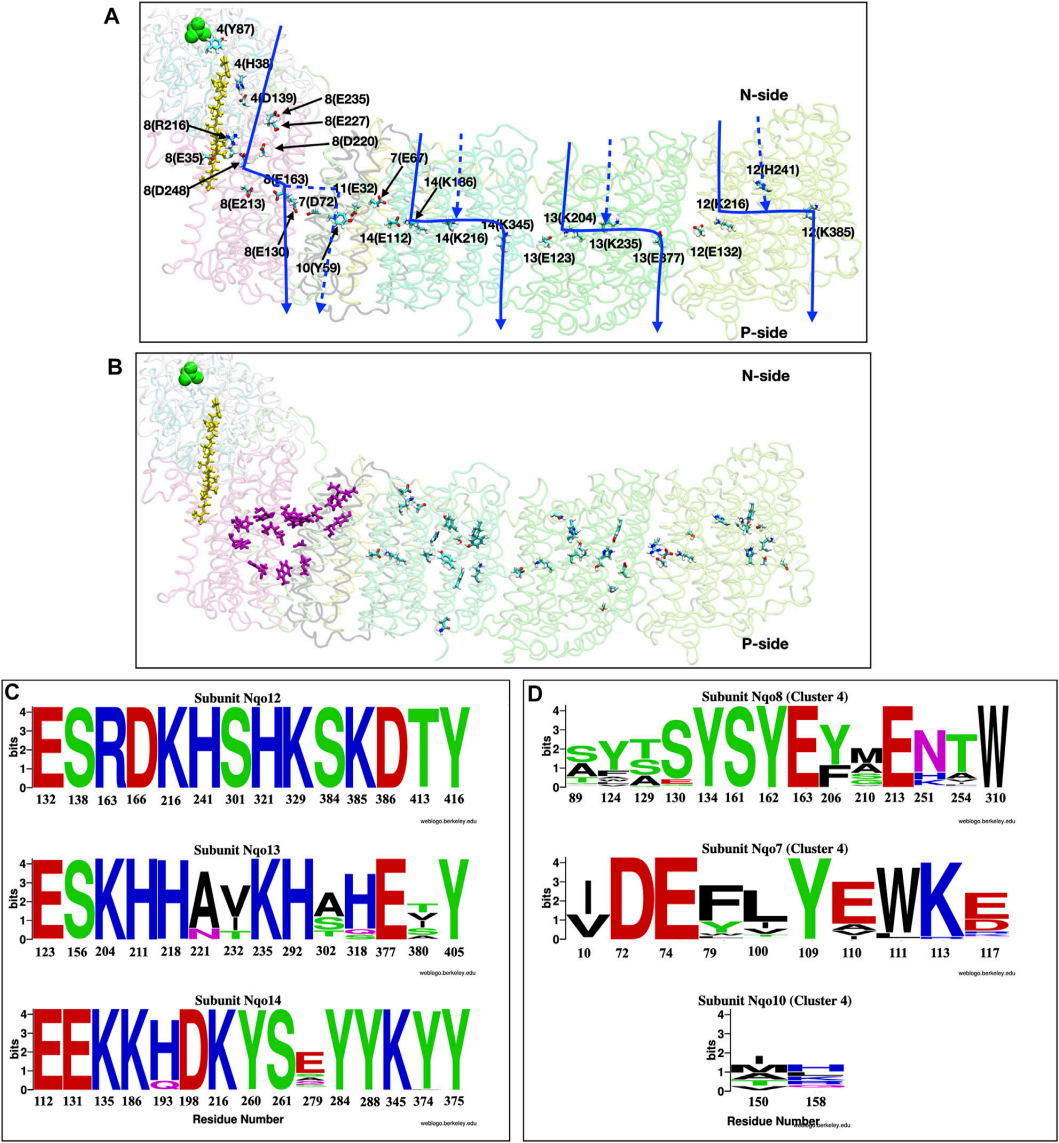
**(A,B)** Structure of the full membrane domain of complex I and only the Nqo4 subunit of the periplasmic domain from *T. thermophilus* [PDB ID: 4HEA (Baradaran et al., 2013)] highlighting proton transfer paths. **(A)** Residues that have been proposed to be important for proton pumping are shown as sticks and labeled as subunit number (one letter amino acid designation-residue number). Quinone is yellow and N2 of each Iron Sulfur cluster is green. Approximate locations of putative proton transfer paths are blue arrows and dashed lines show alternative, proposed paths. **(B)** Residues that are included in the conservation analysis. Purple sticks: E channel cluster in the center of the protein [Cluster 4 in (Khaniya et al., 2020)]; Sticks colored by atom type: antiporter residues (Baradaran et al., 2013; Di Luca et al., 2017; Haapanen and Sharma, 2017; Gutiérrez-Fernández et al., 2020). **(C,D)** Conservation analysis shown as Weblogo (Crooks, 2004) representation of multiple sequence alignment of 1,000 complex I sequences (Johnson et al., 2008; Sievers et al., 2011). **(C)** Residues in the three linear antiporter pathways in Nqo12 **(Top)**, Nqo13 **(Middle)** and Nqo14 **(Bottom)** subunits. **(D)** Residues in the E-channel cluster 4. Residues from Nqo8 **(Top)**, Nqo7 **(Middle)** and Nqo10 **(Bottom)**.

The quinone, Q, is often ubiquinone but can be menaquinone in bacteria such as *Thermus thermophilus,* the source of the protein for the first complete crystal structure (Baradaran et al., 2013). The transfer of the two electrons, which occurs in the N-side peripheral arm, leads to the pumping of four protons from the N- to P-side of the membrane embedded portion of the protein (Figure 8).

Complex I combines elements of two disparate proteins. The long, peripheral arm is likely derived from a soluble hydrogenase and three of the proton channels are related to Mrp Na-H antiporters (denoted antiporter channels), however, the fourth, E-channel, is unique to Complex I and its close relatives (Efremov and Sazanov, 2012; Brandt, 2019). Complex I is remarkable, as the tightly coupled electron transfer and proton pumping elements are separated by as much as 300 Å from the NADH binding site, at the end of the peripheral arm, to the distal proton pumping subunit (Baradaran et al., 2013; Kaila, 2018). Quinone binding and reduction lead to a rotation of the soluble arm that connects the redox reactions to proton pumping in some way (Gupta et al., 2020; Gutiérrez-Fernández et al., 2020; Kampjut and Sazanov, 2020).

*Antiporter: simple pathway.* Complex I provides examples of both simple and complex proton transfer pathways. There are four proton paths, three through the antiporter subunits and one through the E-channel (Hirst, 2013; Ripple et al., 2013; Sazanov, 2015; Di Luca et al., 2017; Haapanen and Sharma, 2018; Saura and Kaila, 2019). The crystal structures show likely, linear paths through each antiporter subunit (Efremov and Sazanov, 2011; Baradaran et al., 2013; Zickermann et al., 2015) which have chain of well conserved acidic and basic residues in the center running parallel to the membrane (Figures 8A,B) (Fearnley and Walker, 1992; Torres-Bacete et al., 2007; Efremov and Sazanov, 2012). Recognizable water chains leading to the N- and P-sides are seen in computational studies (Kaila et al., 2014; Di Luca et al., 2017; Haapanen and Sharma, 2017; Röpke et al., 2020). Moving along each pathway from the N-side is a Glu/Lys pair then a central Lys followed by either a Lys or Glu (Baradaran et al., 2013; Kaila, 2018). Their protonation states change as the proton is handed from one ionizable residue to the next. Thus, the antiporter channels are simple linear proton transfer paths.

With a linear proton transfer path, it is often possible to identify a unique gating element. Simulations have been carried out to investigate the behavior of Complex I with different protonation states for these residues (Kaila et al., 2014; Di Luca et al., 2017; Haapanen and Sharma, 2017). Increasing the net charge in the interior leads to water molecules being brought into the protein in MD trajectories and they are expelled when the charges are neutralized (Kaila et al., 2014; Hummer and Wikström, 2016; Di Luca et al., 2017). These hydration/dehydration changes will gate proton transfer through the channels, similar to that described above for Asp96 in bR. They can be validated by seeing waters in different locations in structures trapped in different intermediates or by interpretation of IR spectroscopy (Lórenz-Fonfría et al., 2008). However, a buried charge will attract water in MD simulations so it is important that the residue protonation states be correctly assigned in the simulation (Hummer and Wikström, 2016).

Comparison of the structures of Complex I from different organisms shows conservation of the P-side proton release paths in the antiporter subunits. However, on the N-side MD studies (Kaila et al., 2014; Di Luca et al., 2017; Haapanen and Sharma, 2017) found a pathway similar to one identified in the crystal structure of *Y. lipolytica* (Zickermann et al., 2015) Complex I but different from the one proposed from the *T. thermophilus* (Sazanov, 2015) crystal structure. Thus, it is not known if the exit path is conserved. Similar changes in pathways through evolution are also found comparing A- and B-type CcO as will be described below.

*E-channel: A complex proton transfer path.* In contrast to the linear proton transfer path seen through the three antiporter subunits, the fourth proton travels through a path directly under the periplasmic arm denoted the E-channel. This region has a web of water molecules and polar and protonatable residues characteristic of a complex proton transfer path with several PLS clusters (Di Luca et al., 2017; Saura and Kaila, 2019; Gutiérrez-Fernández et al., 2020; Khaniya et al., 2020). There are several competing proposals for the proton transfer path through the E-channel. It has been suggested to use subunits Nqo10 and Nqo11 (Efremov and Sazanov, 2011; Zickermann et al., 2015) or subunit Nqo8 (Baradaran et al., 2013). Various computational studies also provide different answers (Kaila et al., 2014; Di Luca et al., 2017; Haapanen and Sharma, 2017). This uncertainty about the route is characteristic of complex proton transfer paths. There is a growing consensus that residues in subunits Nqo7, 8 10 and 11 are important for E-channel function (Figure 8A). Network analysis, which can accommodate complexity, has proposed a complete path through subunit Nqo4 and Nqo8 at the N-side entry, moving through subunit Nqo8 and Nqo7 in the center, and exiting through subunit Nqo10 and Nqo11 (Khaniya et al., 2020).

*Role of quinone in Complex I as a gate.* Complex I is able to couple the energy releasing redox reactions in the peripheral arm to the energy requiring proton pumping through four, distant well separated pumping sites (Baradaran et al., 2013; Kaila, 2018). The quinone binding site in complex I is ≈25–30 Å above the membrane surface, which is different from its location in any other quinone dependent membrane protein (Baradaran et al., 2013; Zickermann et al., 2015). Quinone binding leads to the rotation and tilt of the peripheral arm (Gutiérrez-Fernández et al., 2020). MD simulations (Gamiz-Hernandez et al., 2017; Warnau et al., 2018; Gupta et al., 2020) and Monte Carlo sampling (Khaniya et al., 2020) find changes in the connectivity of the hydrogen bond network that depend on the presence and redox state of the quinone. Movement of subunit Nqo4 and Nqo8 leads to changes in the E-channel hydrogen bond network that propagate into the first antiporter channel by a distortion near Nqo10 (Tyr 59) (Gutiérrez-Fernández et al., 2020). The shifts in hydrogen bond network and residue protonation initiated by the quinone reactions thus yield changes in the interaction between the key Glu/Lys residues in the very distant antiporter channels (Efremov and Sazanov, 2011; Kampjut and Sazanov, 2020). This web of long-range communication is not needed in smaller proteins such as CcO, described below, where the change in electrostatic potential due to the redox reactions can directly modify PLS proton affinity coupled to proton pumping.

MD trajectories show quinone binding influences the E-channel by enriching the number of hydrogen bonds near the N-side, which are proposed to open the channel for proton uptake (Gupta et al., 2020; Khaniya et al., 2020). However, when QH_2_ is bound the charge of conserved residues change leading to modification of the water wires in the proton transfer channels (Gamiz-Hernandez et al., 2017; Kaila, 2018). The E-channel central region has extended clusters of protonatable residues including Nqo7 (Asp 72), 8 (Glu 130), 8 (Glu 163), 8 (Glu 213) (Khaniya et al., 2020) [the nomenclature uses residue numbering from the *T. thermophilus* complex I in the form as Nqo subunit (residues)].

Beyond the central cluster of polar residues there is a hydrophobic barrier that blocks the proton transfer to the P-side in the E-channel. Thus, while there have been several studies of the proton entry, connections are rarely drawn from the center to the P-side (Baradaran et al., 2013; Kaila et al., 2014; Zickermann et al., 2015; Di Luca et al., 2017; Haapanen and Sharma, 2017). Network analysis of the hydrogen bonds made in MD trajectories suggests several paths that rely on transient wetting events (Khaniya et al., 2020). One lies near Nqo7 (Tyr 7) and 8 (Tyr 124). Another possibility is from Nqo8 (Glu 130) to 10 (Tyr 59) (Gutiérrez-Fernández et al., 2020; Steiner and Sazanov, 2020). However, these proposed bridging residues are not well conserved. Thus, what permits the proton to cross the hydrophobic barrier, and whether it conserved through Complex I evolution, is still an open question. A similar hydrophobic barrier is also seen in the voltage-sensing domain (VSD) of voltage-gated ion channels. Here mutations of the hydrophobic residues make the system leaky, showing the importance of non-polar residues to block uncontrolled proton transfers (Banh et al., 2019).

*Residue conservation.* The conservation and sensitivity of residues to mutation can provide evidence that there is a unique pathway for protons. The multisequence alignment of the residues in the linear paths through the three antiporter subunits (Baradaran et al., 2013; Di Luca et al., 2017; Haapanen and Sharma, 2017; Gutiérrez-Fernández et al., 2020) were compared with that found for the central cluster in the complex E-channel pathway (cluster 4 residues) (Khaniya et al., 2020). The WebLogo (Crooks, 2004) provides a graphical comparison of the results (Figures 8C,D). The residues along the linear antiporter paths are highly conserved. In contrast, the E-channel central cluster shows much weaker conservation, suggesting that, while the cluster as a whole must function, individual residues may not be uniquely important.

*Mutation* The sensitivity to mutation may also distinguish linear from complex proton transfer paths. Thus, mutation of residues along a linear path should severely impair activity. In contrast, a complex path may be less sensitive as there are multiple routes for the proton, though as shown above for bRCs, not all need be equally favorable. Many of the residues in the well-defined antiporter channels have been subjected to site-directed mutations. Mutation of the residues shown in (Figure 8A) severely reduce quinone oxidoreductase activity that is tightly coupled to proton transfer since there is no alternative paths for proton transport (Torres-Bacete et al., 2007; Euro et al., 2008; Michel et al., 2011). However, the E-channel is more complex and there is less consensus about the path. Mutations of proposed E-channel residues often modify but do not kill activity (Taylor et al., 2002; Yang et al., 2009).

## Cytochrome c Oxidase

**FIGURE 9:**
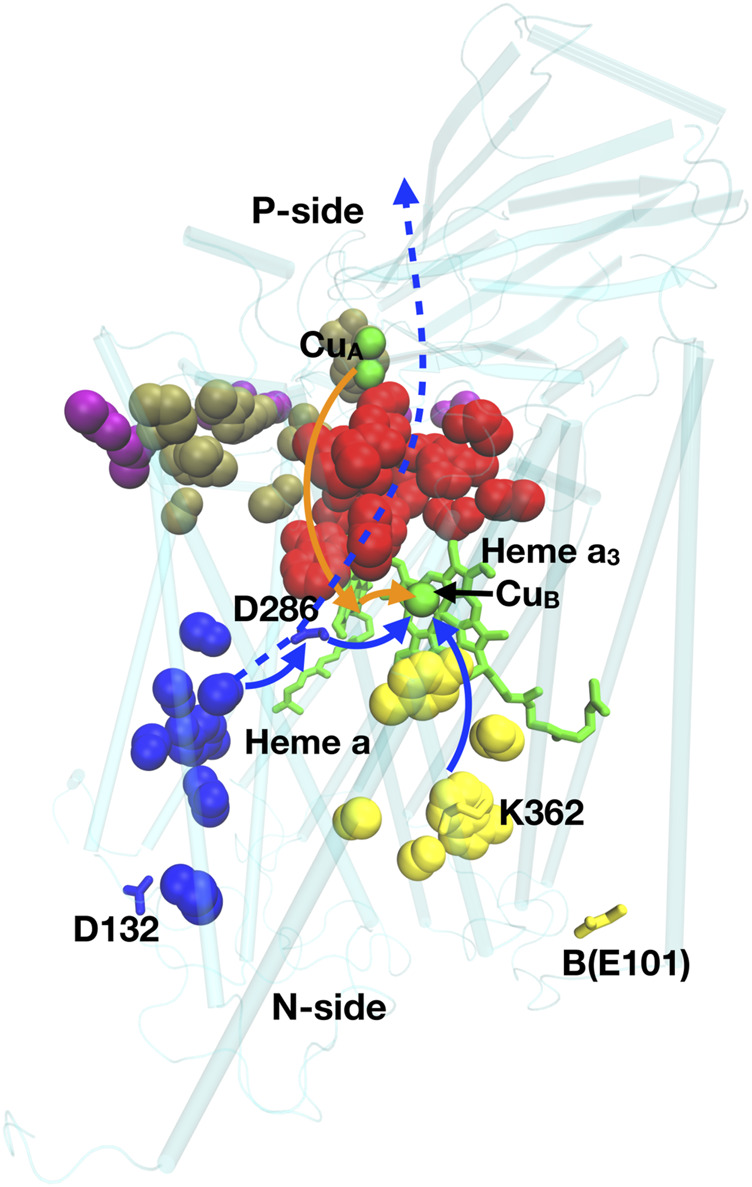
Residues of importance for proton pumping in CcO. Residues D132, K362, D286, B(E101) are shown; heme a and a_3_ are green sticks; Cu_A_ and Cu_B_, are green spheres. Blue spheres: D-channel; red: P-side cluster PLS; yellow: K-channel; Brown: P-exit path; Purple: P-exit surface cluster. Solid blue lines: path of protons used for chemistry in the BNC (heme a_2_ and Cu_B_), dashed blue line: path for pumped protons; orange line: electron transfer path. Structure of *Rb. sphaeroides* CcO from PDB ID:1M56.

CcO is a proton pump belonging to the heme-copper oxidase superfamily (Kaila et al., 2010; Liang et al., 2017; Kaur et al., 2019). The energy for proton pumping comes from electrons from cytochrome c (Cyt c) reducing O_2_ to water in the binuclear (Heme & Cu) center (BNC), located in the protein center (Figure 9). The BNC is reduced stepwise, one at a time to store four electrons. O_2_ reduction takes place in one step in the fully reduced BNC (Kaila et al., 2010; Cai et al., 2020). O_2_ production likewise takes place in one step in the fully oxidized OEC of PSII. This mechanism protects against the release of toxic reactive oxygen intermediates. The overall reaction is:

$${4\text{cyt c}}_{\text{P}}^{3+}{\;+\text{ O}}_{2}\;+\;\left(4+\text{ m}\right){\text{ H}}_{\text{N}}^{+}\;\to {\;4\text{cyt c}}_{\text{P}}^{2+}{\;+\;2\text{H}}_{2}\text{O }+\;\left(4+\text{ m}\right){\text{ H}}_{\text{P}}^{+}$$

Four electrons come from the cytochrome c on the P-side, and four protons from the N-side to the BNC for chemistry. m is the number of protons pumped across the membrane. There are several related classes of CcO, denoted A, B and C. The A-type CcO is found from bacteria to mammals, while B- and C-type are found in bacteria that live at low O_2_ levels. In the A-type CcO, m = 4. B- and C- type CcOs differ in the types of heme used and in the number of protons pumped/electron, with m generally less than four (Lee et al., 2012). CcO provides examples of simple and complex proton transfer paths and simple and cluster PLS as well as a gate generated by hydration/dehydration changes.

*Proton transfer paths through CcO.* In the A-type CcO two linear water filled channels (D- and K-channels) are seen. Each has an essential ionizable residue at the entry on the N-side and at the end near the BNC, but none within the channels. The D-channel has Asp 132 at the entry and the essential, isolated Glu 286 as the PLS (Wikström et al., 2000; Brändén et al., 2001). The K channel has Lys 362 near the BNC and B-Glu 101 near the entry (Ma et al., 1999; Brändén et al., 2002). The buried Glu and Lys are both isolated in hydrophobic parts of the protein. In the ground states, their proton affinity has shifted so both are neutral (*Rb. sphaeroides* CcO numbering used here).

The D-channel carries six of the eight protons in the A-type CcO reaction cycle, while the K-channel carries two. At the center of CcO is heme a and heme a_3_ and Cu_B_ of the active site BNC. As the retinal does in bR, the large cofactors may help to block proton transfer through the protein. The protons from the K channel exit into the BNC to be added to the reduced product water, while the D channel exits between the two hemes. The importance of these linear pathways were demonstrated by mutation of the residues at the beginning and end of the channels leading to loss of activity (Jünemann et al., 1997; Qian et al., 1997; Mills and Ferguson-Miller, 2002). Oddly, the D-channel is missing in B- and C-type CcOs, with only a K-channel remaining (Lee et al., 2012). Thus, as suggested in complex I, proton transfer paths may shift through evolution. In the B- and C- type CcOs it remains unclear how the pumped protons move around the active site to be delivered to the P-side.

*Complex proton transfer pathways*. The P-side of all CcOs has a tangled cluster of strongly interacting polar and protonatable residues that do not provide an obvious single exit path, although linear paths have been suggested (Popović and Stuchebrukhov, 2005; Björck et al., 2019). The hydrogen bond network on the P-side of A- and B-type CcO, was analyzed using Monte Carlo sampling and network analysis (Cai et al., 2018; Cai et al., 2020). Calculations were initiated with experimental crystal structures as well as with snapshots from MD trajectories carried out in different redox states of the hemes and protonation states of key residues. This analysis recognized a linear proton transfer path through the D-channel in the A-type CcO. A very large cluster of interconnected residues was identified as the P-side PLS (Figure 9). This cluster exits through several paths to a region near the cytochrome c binding site. Thus, there is an exit region not a unique exit for protons in this complex path.

*Single residue PLS in CcO.* Glu 286 is located at the top of the D-channel of A-type CcO (Kaila et al., 2010). It is isolated from other protonatable residues so forms a simple PLS. It plays an essential role, releasing a proton to the BNC for chemistry and to a P-side PLS cluster for pumping. The X-ray crystal structures show it is in a dry region and all simulation techniques give it a high proton affinity as there is nothing in the structure to stabilize an anionic residue (Hummer and Wikström, 2016). Measurements found a pK_a_ of ≈10 for turnover that is assigned to Glu286 (Namslauer et al., 2003). However, MD simulations showed protonation of a propionic acid in the P-side PLS breaks a hydrogen bond and opens a cavity which then fills with water (Goyal et al., 2013; Son et al., 2017).

The hydration of the water cavity near Glu 286 also serves as a gate for the proton transfer pathway. In the crystal structures and in protein equilibrated in MD trajectories without the water cavity there is no exit found from the D-channel to the P-side PLS. Thus, the closed cavity blocks the backflow of protons (Cai et al., 2018). However, when the cavity is hydrated Glu 286 becomes well connected to the extensive PLS cluster on the P-side. Changes in hydration also help control the proton transfer from Glu 286 to the BNC (Wikström et al., 2003) Thus, hydration tunes the proton affinity of an isolated residue and serves as a gate in the proton transfer pathway, a pattern described for Asp 96 in bR and for the antiport subunits in complex I.

*The role of tautomer shifts in a complex PLS.* A proton pump must regulate the thermodynamics of PLS loading and unloading, and then change proton affinity when the reaction progresses. This requires tuning the free energy difference between the loaded and unloaded states at the pH of interest as well as the shift in this value as the protein goes through the reaction cycle (See fuller description in Supplementary Material S1). The PLS must remain in the appropriate loaded/unloaded state until the reaction has progressed and the accessibility of N- and P-side is modified by the gates opening/closing (Kim et al., 2007; Kim et al., 2009; Kim and Hummer, 2012; Stuchebrukhov, 2018; Stuchebrukhov, 2019). The challenge is to find the changes that can trap, hold and then release the proton. An analysis of the proton distribution in the PLS cluster in B-type CcO provides some insight into the atomic details of one mechanism.

**FIGURE 10:**
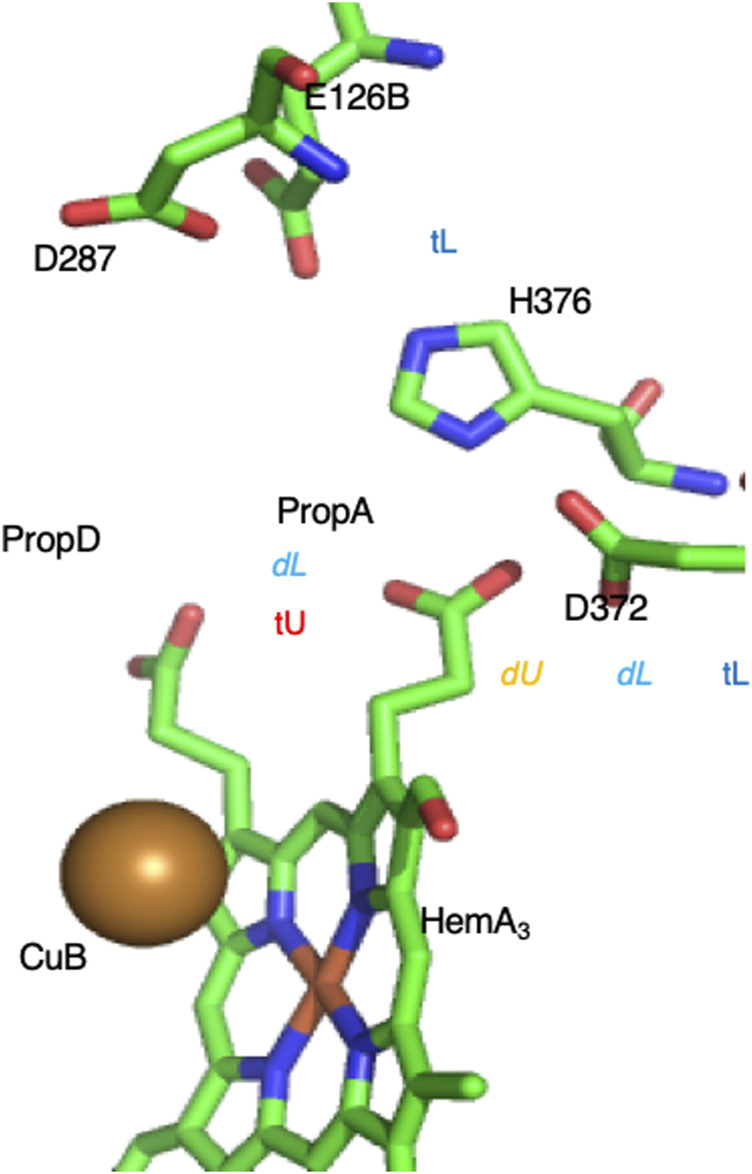
Complex PLS on the P-side of the B-type CcO. The proton moves between Prop A [trapped unloaded (tU), with very low proton affinity] to D372 [dynamic unloaded (dU)] where electron transfer to Heme A_3_ or CuB in the BNC active site 15 Å distant will lead to proton loading into the cluster (Cai et al., 2020). In the loaded state one proton is on D372. If the second proton is on PropA the system is dynamic (dL), so addition of a proton to the product water trapped in the BNC leads to the PLS unloading. If the second proton is on H386 the cluster proton affinity is too high to lose a proton (tL). The intra-cluster distances determine the relative energy of the loaded and unloaded tautomers. The crystal structure is likely trapped in the loaded state. Structure of *Th. thermophilus* CcO from PDB ID:3S8F.

On the P-side of the B-type CcO, an extended cluster of six residues was found to behave as a PLS (Figure 10) (Cai et al., 2020). The unloaded PLS has one proton bound (net charge -4) while the loaded cluster has two protons. The protein surrounding the PLS provides sufficient long-range positive potential to stabilize the cluster negative charge. There are six tautomers with one proton and 12 with two protons distributed over the six residues (Eq. 1). By investigating the proton affinity of different tautomers in snapshots derived from MD trajectories it was found that a shift in the hydrogen bond pattern changes the tautomer selected. This resulted in dynamic states where a loaded and unloaded state are close in energy so changes at the active site lead to proton binding or release. However, the BNC is ≈15 Å from the PLS and the change in the BNC do not shift the PLS proton affinity enough to fully load a proton to the PLS cluster. This behavior is seen Supplementary Figures S2A,B when there is only a small shift in proton affinity in a group with a pK_a_ near the pH (Supplementary Figure S2C, middle titration). The problem of incomplete loading/unloading appears to be solved by moving the bound protons and rearranging the hydrogen bonding pattern, which is described as a tautomer trap. This leads to the PLS being trapped loaded or unloaded because the cluster proton affinity is either too high or too low (Supplementary Figure S2C, right-most titration) or unloaded (Supplementary Figure S2C, left-most titration).

The proton shift in the CcO complex PLS shows how a tautomer trap can solve the problem that a PLS, which is sensitive to changes in the protein, may not strongly trap the proton. Thus, in active structures, the change in proton affinity due to the BNC will lead to some changes in the PLS protonation state. But this shift in free energy is insufficient to reliably move the PLS between being fully loaded and unloaded (Supplementary Figure S2). Then a tautomer shift moves the PLS from the dynamic configuration (where the proton was bound or lost) to the stable, fully loaded or unloaded locked configuration. This mechanism may be similar to activation and inactivation process in voltage gated ion channels, where conformational changes occur when the channels are an active open state, then block the channel and transit to inactive state (Aldrich, 2001). A tautomer trap is only available to a PLS cluster, it is not possible in a single residue PLS.

## Conclusion

The structure of multiple proton transfer paths in several proteins that add to the transmembrane electrochemical gradient show a range of motifs. Thus, they can be simple linear paths as found in the D- and K- channels of CcO and the antiporter subunits of complex I. They can also be complex paths as seen on the P-side of CcO and the E-channel of complex I. Paths can be filled with water so that a proton never needs to use a side chain as found around the OEC in PSII or to be handed through a mixture of side chains and water molecules as found in GFP and in bRCs. With simple, single site PLS, as at the exit from the D-channel and the N-side of bR, changes far from the PLS trigger water influx that leads to proton release and production of a water chain to ferry protons. A similar mechanism is used in the antiporter subunits of complex I. In contrast, in the PLS cluster on the proton release side of bR and CcO, small, local rearrangements of a cluster of strongly interacting residues leads to large changes in proton affinity to cause the PLS to load and unload.

Thus, the framework that proteins will have proton transfer paths, Proton Loading Sites (PLS) and gates allows the analysis of each of these proteins. However, the motifs vary in the residues that make up the needed elements and in their complexity. Each proton pump reviewed here use structures with different complexity for different parts of the proton transfer paths. The advantages of different motifs remain to be determined.
