# Supplementary material for: Protein Motifs for Proton Transfers That Build the Transmembrane Proton Gradient
Source: Front Chem. 2021 Jun 15;9:660954. doi: 10.3389/fchem.2021.660954 (PMC8239185; doi:10.3389/fchem.2021.660954)
Supplement: Supplementary file 1 [file DataSheet1.zip › Supplementary Material.docx]

Supplementary Material

**SI.1.** Structure of OEC

**SI.2.** PLS must tune its proton affinity so that it can bind and release a proton during the reaction cycle*.*

**SI.1. Structure of OEC.**


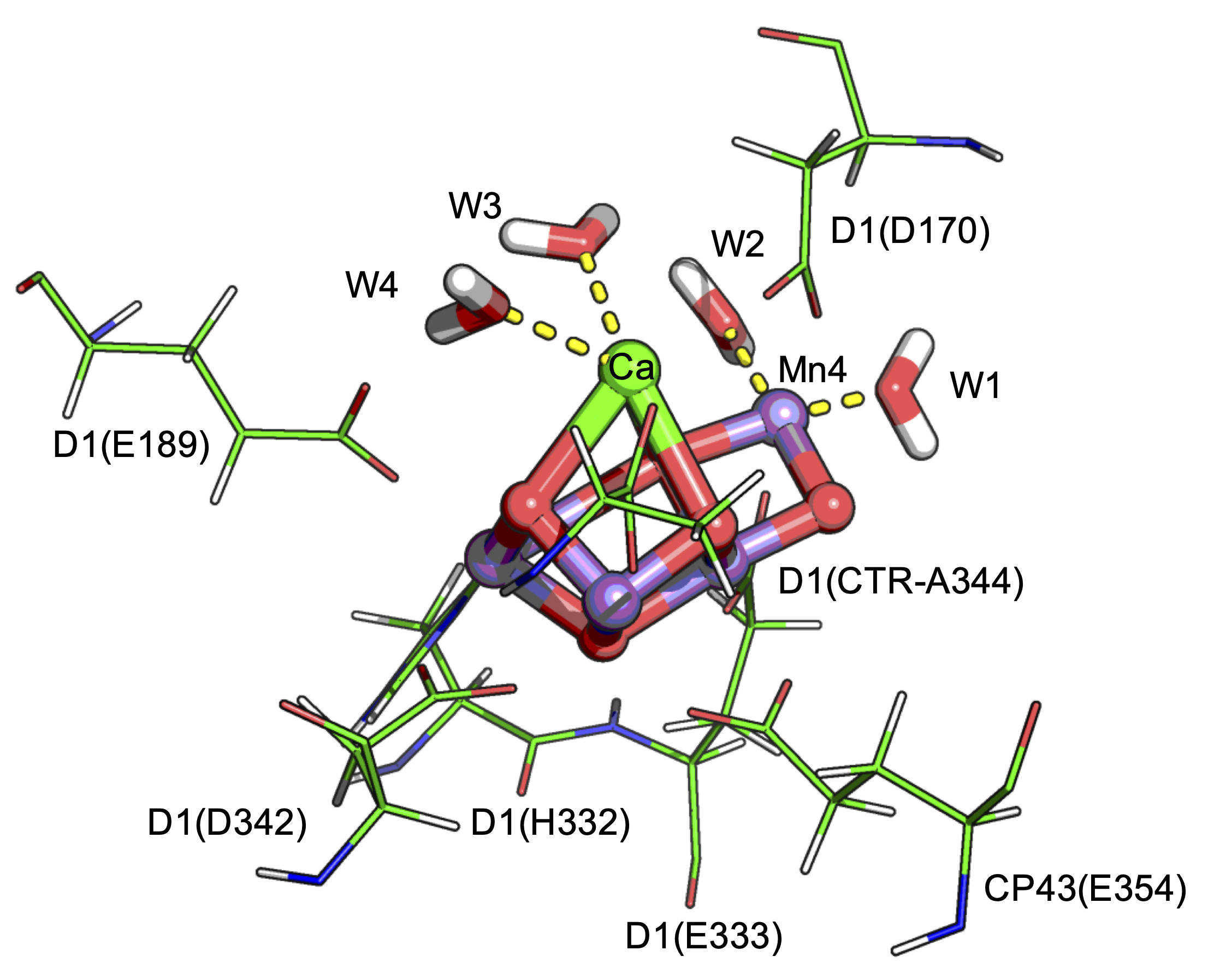


**Figure SI. 1**. The Mn_4_CaO_5_ complex consists of four Mn, one Ca and five O in a distorted cubane structure. Two water molecules are ligated to Ca and two water molecules are ligated to Mn4. There are seven amino acid ligands to Mn4CaO5: D1-D170, E189, H332, E333, D342, CTR-A344, CP43-E354. The figures for the OEC and water channels are from one representative molecular dynamics snapshot of PSII (Kaur et al., 2021).

**SI. 2. PLS must tune its proton affinity so that it can bind and release a proton during the reaction cycle***.*

The underlying rules for a functional PLS are straightforward, but the precision with which the protein must tune the proton affinity to achieve a high proportion of the protein moving between loaded and unloaded states is often not recognized. The takeaway is that the PLS must start with its proton affinity in the right range and that the proton affinity must shift by a large amount to function. We will walk through the rules for a single residue serving as a simple PLS.

The PLS has two states: one that is loaded, with a proton bound, and one that is unloaded, with the proton released. The free energy difference between the loaded (G_L_) and unloaded (G_U_) PLS in a given reaction intermediate at a given pH is:

∆G_LU_^pH^ = ∆G_L_^pH^ - ∆G_U_^pH^ (1)

This is true for an individual residue or a PLS cluster. For a single residue with a well-defined pK_a_:

∆G_LU_^pH^ = c (pH - pK_a_) (2)

where c defines the units, e.g., at 298K, c=1.36 kcal/mol, 59 meV (Gunner et al., 2020). ∆G_LU_ = 0 when the pH = pK_a_. The PLS will be loaded when the pH < pK_a_ (∆G_LU_ negative). Given the relative energy of the two states the probability of the PLS being protonated at a given pH, N_L_^pH^_,_ is obtained using the relationship from statistical mechanics:

$N_{L}^{pH}=\frac{{10}^{{-\Delta G}_{L}^{pH}}}{{10}^{{-\Delta G}_{L}^{pH}}+{10}^{{-\Delta G}_{U}^{pH}}}$ (3)

Proton loading relies upon the PLS proton affinity, ∆G_LU_, changing as the protein moves through the reaction cycle. The affinity should be high (negative ∆G_LU_) when a proton should be loaded into the PLS and low (positive ∆G_LU_) when it needs to be released.

∆∆G_LU_ = (∆G_LU_^loaded^ - ∆G_LU_ ^unloaded^) (4)

For a single residue, with a simple titration curve:

∆∆G_LU_ = -c (pK_a_^loaded^ - pK_a_^unloaded^) (5)

Figure 1 shows how the shift in proton affinity between reaction intermediates, ∆∆G_LU_, as well as the absolute proton affinity ∆G_LU_ determines the proton uptake stoichiometry. Thus, Figure 1(B) shows that when ∆∆G_LU_ shifts the pK_a_ by 4 pH units (5.44 kcal/mol), there is a narrow pH range where almost one is proton taken up by the PLS. Smaller shifts in affinity lead to less proton binding, over a smaller pH range. As the shift in proton affinity between loading and unloading intermediates increases, the pH range over which protons are bound becomes wider so the binding of a proton will be less sensitive to the exact pH or small changes in PLS proton affinity. Thus, the proton affinity needs to change by >5.4 kcal/mol for all PLS in a protein sample to bind and release a proton. Smaller ∆∆G_LU_ will result in a lower stoichiometry of protons pumped.

*Additional constraints on a functioning PLS.* A PLS must change ∆G_LU_ from high proton affinity to low affinity to cycle between loading and unloading. However, if the surroundings tune the PLS proton affinity to be too low (pK_a_ too low, ∆G_LU_ too positive), the changes induced by the reaction) cannot increase the proton affinity enough to bind the proton; likewise, if the background proton affinity is too high (∆G_LU_ too negative) a positive ∆∆G_LU_ may not be sufficient to cause a proton to be bound. Thus, while all protonatable residues can feel changes in proton affinity due to the reaction, most keep their protonation states unchanged. A functional PLS is poised so both the background proton affinity (∆G_LU_) and the changes induced by the reaction (∆∆G_LU_) are in the right range to have the needed sensitivity to the reaction.

| 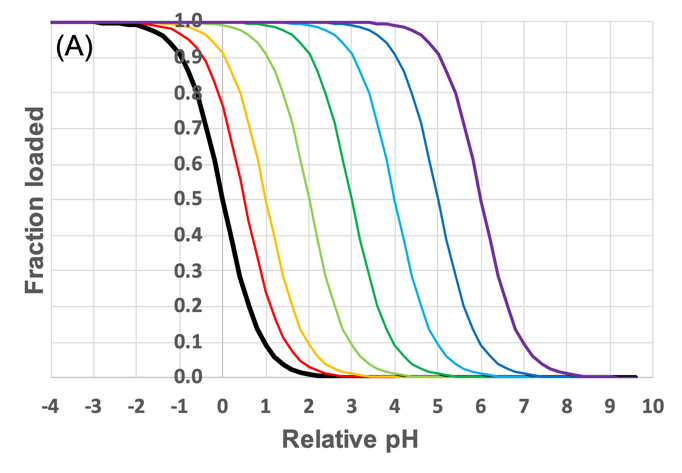 | 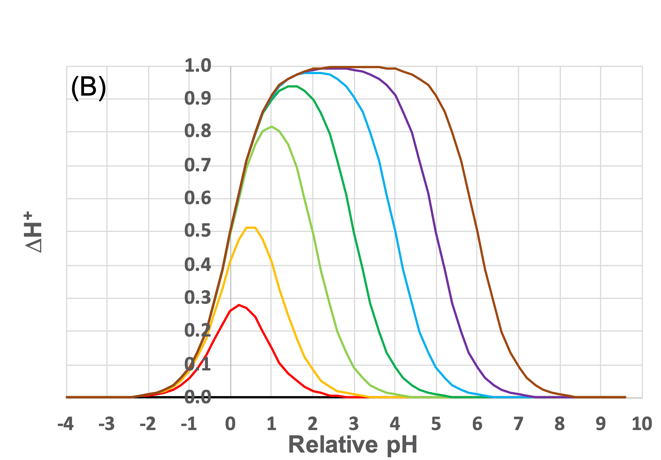 |
| --- | --- |
|  | |

**Figure SI. 2**. A simple PLS, following the Henderson-Hasselbalch relationship (eqn. 3). (A) Titration of the PLS, with different proton affinity. Black: the unloaded state, with the smallest proton affinity. The ‘relative pH’ is zero at the pK_a_ of this titration. Titrations moving to the right shifts the proton affinity by 0.5, 1, 2, 3, 4,5 and 6 pH units. Each 1 pH unit shift represents a ∆∆G_LU_ of 1.36 kcal/mol. (B) The proton uptake for each shift in proton affinity is the difference between the protons bound in the black titration and in each titration with a higher proton affinity. There is a narrow pH range where protons are bound. If the proton affinity is too high relative to the pH (pH << pK_a_), there will be negligible proton release. Likewise, if the proton affinity is too low (pH >> pK_a_), the PLS will never bind. The peak of proton loading is at a pH near the pK_a_ of the unloaded state + 1/2∆∆pK_a_ between the unload and loaded states of the protein. (C) pH dependence of proton binding for three sites with different proton affinities. Insets show a schematic of the relative free energy of unloaded (red) and loaded (blue) states at pH 7. Left: Red titration and associated relative energy sketch: A PLS with a low proton affinity, at pH 7 a modest change in ∆G_LU_ cannot lead to proton binding; Middle: Black titration: ∆G_LU_ is near zero at pH 7, the PLS pK_a_; Modest changes in proton affinity will lead to proton binding/release; Right: Blue titration: High proton affinity. At pH 7 protons are tightly bound and small changes in proton affinity do not lead to proton release.

*PLS clusters.* The PLS and complex proton transfer paths often have regions with many interacting, buried ionizable and polar residues (Lancaster et al., 1996; Kannt et al., 1998). For a PLS cluster with n protonatable residues there are n+1 charge states and 2^n^ microstates, which identify the number and distribution of protons (Gunner et al., 2020). The charge ranges from -N_acids_ (the number of acids) (assuming all bases are neutral) to +N_bases_ (number of bases) (assuming all acids are neutral) + the state where all are neutral. Tautomers are protonation microstates with the same charge but different proton locations. With m protons distributed over n binding sites in a PLS there are:

$\frac{\mathbf{n!}}{\boldsymbol{m!}\left( \boldsymbol{n-m} \right)\boldsymbol{!}}$ (6)

tautomers. ∆G_LU_=0 when the energy of two cluster charge states is the same. The relative energies of the different tautomers determine the proton positions within the loaded and unloaded clusters. The probability of a given tautomer state is:

$\boldsymbol{N}_{\boldsymbol{j}}^{\boldsymbol{pH}}\boldsymbol{=}\frac{\boldsymbol{10}^{\boldsymbol{-\Delta G}_{\boldsymbol{j}}^{\boldsymbol{pH}}}}{\sum_{\boldsymbol{i=all microstates}} \boldsymbol{10}^{\boldsymbol{-\Delta G}_{\boldsymbol{i}}^{\boldsymbol{pH}}}}$ (7)

Reference:

Gunner, M. R., Murakami, T., Rustenburg, A. S., Işık, M., and Chodera, J. D. (2020). Standard state free energies, not pK_a_s, are ideal for describing small molecule protonation and tautomeric states. *J Comput Aided Mol Des* 34, 561–573. doi:10.1007/s10822-020-00280-7.

Kannt, A., Lancaster, C. R. D., and Michel, H. (1998). The Role of Electrostatic Interactions for Cytochrome c Oxidase Function. *J Bioenerg Biomembr* 30, 81–87. doi:10.1023/A:1020563629032.

Kaur, D., Zhang, Y., Reiss, K. M., Mandal, M., Brudvig, G. W., Batista, V. S., et al. (2021). Proton egress pathways surrounding the Oxygen Evolving Complex of Photosystem II. *Biochim Biophys Acta Bioenerg* paper accepted.

Lancaster, C. R., Michel, H., Honig, B., and Gunner, M. R. (1996). Calculated coupling of electron and proton transfer in the photosynthetic reaction center of  *Rhodopseudomonas viridis*. *Biophys. J.* 70, 2469–2492. doi:10.1016/S0006-3495(96)79820-X.
